# Optimizing the delivery of contraceptives in low- and middle-income countries through task shifting: a systematic review of effectiveness and safety

**DOI:** 10.1186/s12978-015-0002-2

**Published:** 2015-04-01

**Authors:** Stephanie Polus, Simon Lewin, Claire Glenton, Priya M Lerberg, Eva Rehfuess, A Metin Gülmezoglu

**Affiliations:** Institute for Medical Informatics, Biometry and Epidemiology, University of Munich, Munich, Germany; Department of Reproductive Health and Research, UNDP/UNFPA/UNICEF/WHO/World Bank Special Programme of Research, Development and Research Training in Human Reproduction (HRP), World Health Organization, Geneva, Switzerland; Global Health Unit, Norwegian Knowledge Centre for the Health Services, Oslo, Norway; Health Systems Research Unit, Medical Research Council of South Africa, Cape Town, South Africa; Department of Community Medicine, University of Oslo, Oslo, Norway

**Keywords:** Systematic review, Contraception, Task shifting, Low income countries, Middle income countries, Effectiveness, Safety, IUD, Tubal ligation, Vasectomy, Injectables

## Abstract

**Objective:**

To assess the effectiveness and safety of task shifting for the delivery of injectable contraceptives, contraceptive implants, intrauterine devices (IUDs), tubal ligation and vasectomy in low- and middle-income countries.

**Methods:**

Multiple electronic databases were searched up to 25 May 2012 for studies which had assessed the delivery of contraceptives by health workers with lower levels of training, compared to delivery by health workers usually assigned this role, or compared to no organized provision of contraceptives. We included randomized controlled trials, non-randomized controlled trials, controlled before-after studies, and interrupted time series. Data were extracted using a standard form and the certainty of the evidence found was assessed using GRADE.

**Results:**

We identified six randomized controlled trials published between 1977 and 1995 that assessed the safety and effectiveness of task shifting for the delivery of long-term contraceptives. Two studies assessed IUD insertion by nurses compared to doctors, two assessed IUD insertion by auxiliary nurse-midwives compared to doctors, one assessed tubal ligation by midwives compared to doctors, and one assessed the delivery of vasectomy by medical students compared to doctors. In general, little or no difference was found in contraceptive outcomes between cadres. Study design limitations and the low number of eligible studies, however, allow only limited conclusions to be drawn.

**Conclusions:**

The findings indicate that task shifting for the delivery of long-term contraceptives may be a safe and effective approach to increasing access to contraception. Further research is needed because the certainty of the evidence identified is variable.

**Electronic supplementary material:**

The online version of this article (doi:10.1186/s12978-015-0002-2) contains supplementary material, which is available to authorized users.

## Introduction

Increasing people’s access to modern contraceptive methods is seen as a highly effective way to protect the health and well-being of women and children [1,2]. However, the global shortage of health workers has had a severe impact on family planning services. In many settings, human resource constraints limit access to contraceptives, making it difficult for people to make informed and free choices about family planning. In some low- and middle-income countries (LMICs), for example, the unmet need for family planning affects 30% or more of the population [3]. It is estimated that 86 million unintended pregnancies every year are caused by inadequate access to family planning services [4].

Task shifting is one potential strategy to address the problems associated with the shortage and maldistribution of health workers and to help achieve the UN Millennium Development Goals 4 and 5 for maternal and child health [5,6]. Task shifting – or task sharing – is defined by the World Health Organization (WHO) as “the training of cadres who do not normally have competencies for specific tasks to deliver these tasks and thereby increase levels of health care access” [7]. Increasing the number of health workers who deliver contraceptives is a key component of increasing access to contraceptive methods, and in many LMICs task shifting has been used for decades to achieve this [8]. Task shifting initiatives may be particularly relevant when increasing access to contraceptives among vulnerable groups such as young and poor women, unmarried women, migrants, and those living in rural areas.

But the provision of contraceptive services by health workers with lower levels of training may have adverse effects if, for example, the advice or the delivery of the care is inappropriate. Ineffective delivery of a particular method may also fail to improve access to contraception. Evidence is therefore needed to ensure that task shifting is both safe and effective. Several systematic reviews have examined the safety and effectiveness of task shifting for a range of maternal and child health interventions [9-12]. In 2012, Janowitz et al. [8] produced an overview of studies evaluating task shifting interventions of various contraceptive methods. Some of the included studies used cohort and case control designs. Other reviews have assessed the effectiveness of using lay health workers (LHWs) to provide injectable contraceptives [13]. The reviews produced thus far, however, have either not assessed the safety and effectiveness of the delivery of a wide range of contraceptives by a range of health workers, or have been based on study designs that cannot provide a rigorous assessment of the safety and effectiveness of the interventions described.

Our review forms part of a series of studies intended to inform the World Health Organization’s ‘Recommendations for Optimizing Health Worker Roles to Improve Access to key Maternal and Newborn Health Interventions through Task Shifting’ (OPTIMIZEMNH) [7]. The objective of this systematic review is to assess the effectiveness and safety of task shifting in the delivery of injectable contraceptives, contraceptive implants, intrauterine devices (IUDs), tubal ligation and vasectomy in LMICs.

## Methods

Methods endorsed by the Cochrane Collaboration [14,15] were used when conducting this review.

### Criteria for selecting studies

#### Types of studies

The review included randomized controlled trials (RCTs), non-randomized controlled trials (NRCTs), controlled before-after studies (CBAs), and interrupted time series (ITSs). For further information about these study types, as defined by the Cochrane Effective Practice and Organization of Care (EPOC) Group [14], please see Additional file 1.

#### Types of interventions

We included studies of the insertion or removal of an intrauterine device (IUD) or contraceptive implant; the provision of a contraceptive injection with a standard syringe or with an auto-disabled, pre-filled injection device (CPAD) such as Uniject™; or the performance of tubal ligation or vasectomy.

We included studies in which the interventions were delivered by lay health workers, auxiliary nurses, auxiliary nurse-midwives, nurses, midwives, associate clinicians or non-specialist doctors. Studies of medical students were also included. This is because students have minimal training and experience in contraceptive delivery and are therefore effectively similar to less specialized cadres or to cadres with lower levels of training than doctors. All the cadre definitions in this review are based on those specified in the OPTIMIZEMNH recommendations [7].

#### Comparisons

We included studies that compared the intervention to usual care (i.e. the standard approach for delivering contraceptives in the studied setting), including:

(i) The delivery of contraceptives by the health workers usually assigned this role; or
(ii) No organized system of contraceptive provision.

#### Types of participants and settings

We considered studies in which the contraceptive methods were delivered to men or women of reproductive age. We limited the review to studies undertaken in LMICs, as the focus of the WHO guidelines was on these settings because they have the greatest burden of unmet contraceptive needs [3].

#### Types of outcomes

We included studies that assessed outcomes related to the individual or medical safety and effectiveness of task shifting for contraceptive delivery. We also considered the effectiveness of task shifting for contraceptive delivery at a population level in terms of contraceptive coverage, which we defined as contraceptive uptake rates in a population. These outcomes were selected in order to address the main review question on the safety and effectiveness of task shifting for contraceptive delivery. Other reviews of the literature that also informed the WHO guidance looked at barriers and facilitators to implementing task shifting [16]. Table 1 provides an overview of the primary outcomes for the review and how they were defined for each contraceptive method. Patient satisfaction with the intervention was included as a secondary outcome.

**Table 1:** **Overview of primary outcomes for each contraceptive method considered in the review**

***Outcome category***	***Contraceptive method***			
	**Injectable contraceptive**	**Contraceptive implants**	**IUDs**	**Tubal ligation/vasectomy**
**Safety and effectiveness of contraceptive delivery**	Complication rates during provision of the injection	Complication rates during insertion/removal (e.g. damage of vessels, tissues)	Complication rates during insertion/removal (e.g. perforation, pain during insertion of IUD)	Complication rates during procedure
		Insertion/removal failure rates	Insertion/removal failure rates	Tubal ligation: Duration of operation*
	Post-procedure complications (e.g. haematomas, infection rate)	Post-procedure complications (e.g. removal rate, infection rate)	Post-procedure complications (e.g. expulsion rates, infection rates, removal rates)	Post-operative complications (e.g. infection rate)
			Referral rates: during IUD insertion, or after IUD insertion*	For vasectomy: oligospermia rates*
	Unintended pregnancy rates			
**Coverage**	Uptake of contraceptives by intended recipients			
	Contraceptive continuation rates			Not applicable

*Included as an outcome after the protocol was finalised.

#### Excluded studies

Studies assessing the delivery of injectable contraceptives by lay health workers were excluded from this review. This is because an evaluation of this specific method of contraception delivery by this health worker group has already been assessed in a systematic review prepared as part of other work informing the WHO OPTIMIZEMNH guidance [17].

Studies examining task supplementation or combinations of task shifting and task supplementation were excluded from this review. Task supplementation refers to instances in which a health worker with a lower level of training supplements or extends the care of a health worker who has a higher level of training by providing a new primary care service. A nurse, for example, may be added to a healthcare team to assist a doctor who performs tubal ligations [9]. The aim of task supplementation, however, is not primarily to address workforce *shortages* but rather to enhance the *quality* of care. Studies in which health workers offered counselling or promotion rather than the actual contraceptive services were also excluded as other reviews have examined these topics [10].

Within the WHO guidance process, it was assumed that all health workers are able safely and effectively to distribute condoms and oral contraceptives (once these have been suggested or prescribed) [7]. For this reason, task shifting studies examining these issues were excluded. Studies, which measured only recipient knowledge, attitudes or intentions, were also not considered because the indicators are not useful measures of the effectiveness of contraceptive task shifting interventions. No studies were excluded on the basis of language.

### Search methods

The PubMed, POPLINE, Cochrane Central Register of Controlled Trials, EMBASE, CINAHL, WHOLIS, LILACS, and IMEMR (EMRO), WPRIM (WPRO), AIM (AFRO) and IMSEAR (SEARO) databases were searched up to 25 May 2012. Search strategies incorporated selected index terms and free text terms related to different health worker cadres, contraceptive methods, and study designs (Additional file 2 provides a detailed description of the database search strategies used). The reference lists of relevant studies and reviews were hand searched to check for additional potentially eligible studies.

### Data collection and analysis

One review author assessed the potential relevance of all the titles identified from the initial electronic searches. Two review authors then independently assessed which abstracts were potentially relevant and retrieved and independently assessed full-text versions of the abstracts. Any disagreements were discussed, and if consensus was not reached, a third review author was consulted.

Two reviewers independently assessed the risk of bias in the included studies by applying the standard EPOC Group assessment tool for RCTs, NRCTs, CBA and ITS studies [18]. The two reviewers then independently assessed and extracted data for all the included studies using an adapted, standardized version of the Cochrane extraction form (see Additional file 3) [15]. Discrepancies were discussed until consensus was reached. Study authors were contacted to obtain missing information or clarify details.

One reviewer, aided by a second reviewer, assessed the certainty of the evidence related to the primary outcomes using the Grading of Recommendations Assessment, Development and Evaluation (GRADE) tool [15,19].

Findings for each of the health worker categories are listed separately as each health worker cadre has different professional characteristics and skills.

For dichotomous data, we have presented the results individually and as risk ratios with 95% confidence intervals. When events in each group were reported as percentages, the data were converted to show the number of events [15]. For studies that reported continuous data, we planned to use the standardized mean difference so that data from different trials measuring the same outcome could be combined. However, only one of the included studies reported continuous data in this way, and this data assessment method was therefore not applied. The effect measures reported by the studies were analysed with Review Manager Software version 5.1 [20]. Meta-analyses of the data were possible only for few outcomes due to the limited number of studies within each comparison.

The allocation to intervention and the outcome measurements were performed at the individual level in the included studies and we therefore did not consider there to be unit of analysis issues.

We noted the levels of attrition in the included studies. As very few studies met the inclusion criteria for this review, we did not conduct sensitivity analyses to explore the impact of including studies with high levels of missing data or of excluding studies at high risk of bias.

Data heterogeneity was explored visually by scrutinizing the forest plots and looking at the overlap between confidence intervals around the effect estimates for included studies. Investigation of heterogeneity was limited by the small number of included studies.

## Results

### Results of the search

**Figure 1:**
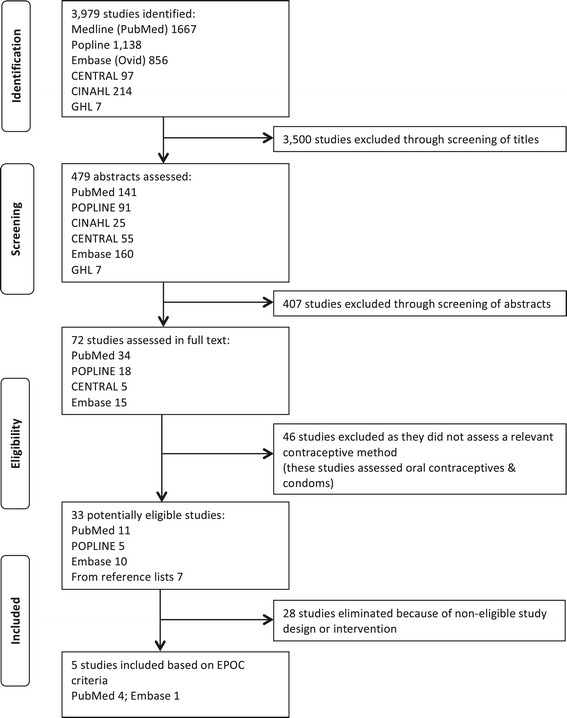
**Flow chart.**

The database searches identified 3,979 potentially relevant records. After screening the titles and abstracts, full text versions of 33 potentially relevant records were retrieved for detailed assessment. Five records reporting six RCTs^a^ met the inclusion criteria of this review (Figure 1). Basic characteristics of included studies are provided in Table 2; more detailed characteristics, including an assessment of the risk of bias in these studies, are described in Additional file 4. Reasons for the exclusion of potentially relevant studies are outlined in Additional file 5. It was not possible to perform a comprehensive risk-of-bias assessment because the data required were not consistently reported in the studies. Most studies were at high risk of bias due to factors such as imprecision of results, dissimilar baseline characteristics, and flaws in sequence generation.

**Table 2:** **Basic characteristics of included studies**

**Author Year**	**Study site**	**Study design**	**Intervention**
Bunyaratavej et al. [25]	Hospital, Bangkok, Thailand	RCT	Vasectomy performed by medical students compared to doctors
Dusitsin et al. [23]	Hospital, Khon Kaen Province, Thailand	RCT	Tubal ligation performed by midwives compared to doctors
Einhorn et al. [24]	Hospital,Bogotà, Colombia	RCT	Insertion of IUDs by nurses compared to doctors
Eren et al. [21] (Study A)	Primary health care setting, Cubuk District, north of Ankara, Turkey	RCT	Insertion of IUDS by auxiliary nurse-midwives compared to doctors
Eren et al. [21] (Study B)	Hospital, Manila, Philippines	RCT	Insertion of IUDS by auxiliary nurse-midwives compared to doctors
Lassner et al. [22]	Hospital, Rio de Janeiro, Brazil	RCT	Insertion of IUDs by nurses compared to doctors

The studies compared the effectiveness of the following contraceptives delivered by the following health worker cadres:

- Insertion of IUDs by nurses compared to doctors
- Insertion of IUDS by auxiliary nurse-midwives compared to doctors
- Tubal ligation performed by midwives compared to doctors
- Vasectomy performed by medical students compared to doctors

The interventions took place in hospitals, with the exception of one study in which the intervention took place in a primary healthcare setting [21] (Study A). The studies were conducted in Rio de Janeiro (Brazil), Bogotá (Colombia), Ankara (Turkey), Manila (the Philippines), and in Bangkok and the Khon Kaen Province (Thailand) between 1977 and 1995.

In the studies of IUD insertion, the nurses and auxiliary nurse-midwives received special training in this procedure. In one study [22], the training of nurses was more specifically described as lasting six to eight weeks and including a theoretical and a practical component, and a minimum of ten solo IUD insertions. In the studies of tubal ligation, midwives were given one year of operating room experience, and training, which lasted twelve weeks on how to perform a tubal ligation [23]. In the vasectomy study, medical students received both theoretical training and practical training, and performed 20 operations under the supervision of a doctor. The number of health workers involved was not always specified but varied between three and 14 health workers in the intervention groups.

The recipients in the included studies were men and women of reproductive age who had no significant reported health pre-conditions. In the studies of IUD insertion by auxiliary nurse-midwives [21] and tubal ligation by midwives [23], some of the female patients received these contraceptive treatments post-partum [21,23] (Study A). None of the other studies [22,24] reported contraceptive delivery postpartum.

The included studies reported the following outcomes for IUDs: contraceptive continuation rates, removal rates, complication rates during insertion, insertion failure rates, expulsion rates, pain during insertion, referral rates and unintended pregnancy rates. For the operative procedures, the included studies reported complication rates during surgery, including duration of operation (one study), and postoperative complications, including oligospermia (one study). None of the included studies assessed contraceptive uptake rates (one of the primary outcomes) or patient satisfaction (one of the secondary outcomes) (see Additional file 4). The full GRADE tables for each comparison are available on request.

#### IUD insertion by nurses compared to doctors (2 RCTs)

IUD insertion by nurses was compared to insertion by doctors in two RCTs [22,24] (see Table 3 Summary of findings).

**Table 3:** **Summary of findings**

**What is the effectiveness of IUD insertion by nurses compared to IUD insertion by doctors?**						
**Patient or population:** patients with IUDs						
**Settings:** Hospital setting, Brazil (Lassner et al. [22]) and Colombia (Einhorn et al. [24])						
**Intervention:** Nurses inserting IUDs						
**Comparison:** Doctors inserting IUDs						
**Outcomes**	Illustrative comparative risks* (95% CI)		**Relative effect**	**No of Participants**	**Certainty of the evidence**	**Comments**
	Assumed risk	Corresponding risk				
	**Doctors inserting IUDs**	**Nurses inserting IUDs**	**(95% CI)**	**(studies)**	**(GRADE)**	
Continuation rates^1^	790 per 1000	782 per 1000	RR 0.99	1786	⊕⊕⊝⊝	
		(743 to 814)	(0.94 to 1.03)	(2 studies)	low^2,3^	
Removal rates^4^	78 per 1000	71 per 1000	RR 0.91	1632	⊕⊝⊝⊝	
		(50 to 100)	(0.64 to 1.27)	(2 studies)	very low^3, 5^	
Complication rates during insertion	17 per 1000	18 per 1000	RR 1.01	1711	⊕⊝⊝⊝	
		(9 to 36)	(0.5 to 2.05)	(2 studies)	very low^3,6^	
Unintended pregnancy rates^7^	12 per 1000	8 per 1000	RR 0.66	1786	⊕⊝⊝⊝	
		(3 to 20)	(0.25 to 1.7)	(2 studies)	very low^2,3,6^	
Insertion failure rate, nulliparous women	34 per 1000	117 per 1000	RR 3.41	263	⊕⊕⊝⊝	
		(40 to 337)	(1.18 to 9.85)	(1 study)	low^2,6^	
Insertion failure rate, multiparous women	9 per 1000	16 per 1000	RR 1.66	1448	⊕⊕⊝⊝	
		(6 to 40)	(0.65 to 4.25)	(1 study)	low^2,6^	
Expulsion rates	54 per 1000	50 per 1000	RR 0.93	1195	⊕⊕⊝⊝	
		(31 to 82)	(0.57 to 1.52)	(1 study)	low^2,6^	
Pain during insertion	108 per 1000	70 per 1000	RR 0.65	1711	⊕⊕⊝⊝	
		(52 to 96)	(0.48 to 0.89)	(1 study)	low^2^	
Uptake of contraceptives - not measured	See comment	See comment	Not estimable	-	See comment	
Referral rates - not measured	See comment	See comment	Not estimable	-	See comment	

*The basis for the **assumed risk** (e.g. the median control group risk across studies) is provided in footnotes. The **corresponding risk** (and its 95% confidence interval) is based on the assumed risk in the comparison group and the **relative effect** of the intervention (and its 95% CI).

**CI:** Confidence interval; **RR:** Risk ratio;

GRADE Working Group grades of evidence.

**High certainty:** We are very confident that the true effect lies close to that of the estimate of the effect.

**Moderate certainty:** We are moderately confident in the effect estimate: The true effect is likely to be close to the estimate of the effect, but there is a possibility that it is substantially different.

**Low certainty:** Our confidence in the effect estimate is limited: The true effect may be substantially different from the estimate of the effect.

**Very low certainty:** We have very little confidence in the effect estimate: The true effect is likely to be substantially different from the estimate of effect.

^1^Continuation rates were measured at 9 months in one study and 12 months in the other study.

^2^Downgraded because of differences in baseline characteristics, including differences in parity and history of pelvic inflammatory disease or sexually transmitted infections.

^3^Downgraded because of high risk of bias in sequence generation and allocation concealment.

^4^In one trial, the outcome was removal rate due to medical reasons and, in the other trial, termination rates due to side effects (including expulsions). Because further information was not provided, it was not clear whether these two outcomes were defined similarly.

^5^Downgraded because studies show different results, one showing no difference between nurses and doctors and the other one showing higher removal rates for nurses than for doctors.

^6^Downgraded because of imprecision (i.e. the confidence interval indicates both benefit and harm or because confidence interval is very wide).

^7^Pregnancy rates were measured at 9 and 12-month follow-ups.

- Both studies assessed **contraceptive continuation rates**. The studies suggest that there may be little or no difference in continuation rates when IUDs are inserted by nurses compared to doctors (RR 0.99, 95% CI 0.94 to 1.03, p = 0.4311, low certainty evidence).
- Both studies examined **removal rates, complication rates during insertion, and unintended pregnancy rates**. It is uncertain whether there are any differences between the performance of doctors compared to nurses for these outcomes because the evidence was of very low certainty.
- One study [22] examined **insertion failure rates**. The study reported different results according to the parity of the woman. For nulliparous women**,** insertion failure rates for IUDs may be higher if inserted by nurses compared to doctors (RR 3.41, 95% CI 1.18 to 9.85, p = 0.0237, low certainty evidence), whereas for multiparous women, there may be little or no difference in insertion failure rates if the treatment is provided by nurses compared to doctors (RR 1.66, 95% CI 0.65 to 4.25, p = 0.2939, low certainty evidence).
- One study [22] examined **expulsion rates**. This study suggests that there may be little or no difference for this outcome when IUDs are inserted by nurses compared to doctors (RR 0.93, 95% CI 0.57 to 1.52, p = 0.7786, low certainty evidence).
- One study [22] examined **pain during insertion** of the IUD and suggested that there may be lower rates of reported pain in the group receiving care from nurses compared to the group receiving care from doctors (RR = 0.65 95% CI 0.48 to 0.89, p = 0.0069; low certainty evidence).
- Neither of the studies measured the uptake of contraceptives or referral rates.

#### IUD insertion by auxiliary nurse-midwives compared to doctors (2 RCTs)

IUD insertion by auxiliary nurse-midwives was compared to insertion by doctors in one paper [21] which described two RCTs: one comparing IUD insertion by auxiliary nurse-midwives and doctors in postpartum women in a hospital in Turkey (Study A), and one comparing the same intervention in women in a primary health care setting in the Philippines (Study B) (see Table 4 Summary of findings). Both studies measured the same outcomes. The studies suggest the following:

**Table 4:** **Summary of findings**

**What is the effectiveness of IUD insertion by auxiliary nurse midwives compared to IUD insertion by doctors?**						
**Patient or population:** patients with IUDs						
**Settings:** Primary health care setting in nine rural villages in Cubuk district, Turkey (Eren et al. [21] Study A) and Jose Fabella						
Memorial Hospital in Manila, Philippines (Eren et al. [21] Study B)						
**Intervention:** Auxiliary nurse-midwives inserting IUDs						
**Comparison:** Doctors inserting IUDs						
**Outcomes**	Illustrative comparative risks* (95% CI)		**Relative effect**	**No of Participants**	**Certainty of the evidence**	**Comments**
	Assumed risk	Corresponding risk				
	**Doctors inserting IUDs**	**Auxiliary nurse-midwives inserting IUDs**	**(95% CI)**	**(studies)**	**(GRADE)**	
Continuation rates^1^	699 per 1000	727 per 1000	RR 1.04	996	⊕⊕⊕⊝	
		(671 to 783)	(0.96 to 1.12)	(2 studies)	moderate^2^	
Removal rates	107 per 1000	115 per 1000	RR 1.08	996	⊕⊕⊕⊝	
		(82 to 162)	(0.77 to 1.52)	(2 studies)	moderate^2^	
Expulsion rates	96 per 1000	81 per 1000	RR 0.84	996	⊕⊕⊕⊝	
		(54 to 121)	(0.56 to 1.26)	(2 studies)	moderate^2^	
Unintended pregnancy rates	20 per 1000	19 per 1000	RR 0.95	996	⊕⊕⊝⊝	
		(8 to 47)	(0.4 to 2.27)	(2 studies)	low^2,3^	
Referral rate during IUD insertion^4^	65 per 1000	52 per 1000	RR 0.80	1058	⊕⊕⊝⊝	
		(33 to 84)	(0.50 to 1.29)	(2 studies)	low^2,3^	
Referral rate after IUD insertion^5^	43 per 1000	64 per 1000	RR 1.49	996	⊕⊕⊝⊝	
		(38 to 109)	(0.88 to 2.54)	(2 studies)	low^2,3^	
Uptake of contraceptives^6^ - not measured	See comment	See comment	Not estimable^6^	-	See comment	
Complication rates at insertion^6^ - not measured	See comment	See comment	Not estimable^6^	-	See comment	
Insertion failure rates^6^ - not measured	See comment	See comment	Not estimable^6^	-	See comment	

*The basis for the **assumed risk** (e.g. the median control group risk across studies) is provided in footnotes. The **corresponding risk** (and its 95% confidence interval) is based on the assumed risk in the comparison group and the **relative effect** of the intervention (and its 95% CI).

**CI:** Confidence interval; **RR:** Risk ratio;

GRADE Working Group grades of evidence.

**High certainty:** We are very confident that the true effect lies close to that of the estimate of the effect.

**Moderate certainty:** We are moderately confident in the effect estimate: The true effect is likely to be close to the estimate of the effect, but there is a possibility that it is substantially different.

**Low certainty:** Our confidence in the effect estimate is limited: The true effect may be substantially different from the estimate of the effect.

**Very low certainty:** We have very little confidence in the effect estimate: The true effect is likely to be substantially different from the estimate of effect.

^1^Continuation rates were calculated from the number of discontinuations at 12 months.

^2^Downgraded because of unclear but potential risk of contamination and inadequate blinding and because of statistical heterogeneity.

^3^Downgraded because of imprecision (i.e. the confidence interval indicates both benefit and harm).

^4^In one study, women were referred because the health worker decided that they were unable to insert the IUD because of postpartum conditions or because they made a failed attempt (i.e. insertion failure). In the other study reasons for referral included: suspected pregnancy, suspected pelvic inflammatory disease, cervicitis and erosion and conditions interfering with IUD insertion (e.g. prolapsed uterus, cervical incompetence).

^5^Where women with IUDs were referred at follow-up visits, because of pregnancy, bleeding problems, suspected pelvic inflammatory diseases (PID), a missing IUD tail, difficulty with insertion or postpartum conditions (anaemia, episiotomy).

^6^The studies did not measure uptake of contraceptives, insertion failure rates or complication rates at insertion.

- There is probably little or no difference in **continuation rates** (RR 1.04, 95% CI 0.96 to 1.12, p = 0.7227, moderate certainty evidence); **removal rates** (RR 1.08, 95% CI 0.77 to 1.52, p = 0.564, moderate certainty evidence); or **expulsion rates** after IUD insertion (RR = 0.84, 95% CI 0.56 to 1.26, p = 0.38, moderate certainty evidence) when IUDs are inserted by auxiliary nurse-midwives compared to doctors.
- There may be little or no difference in **unintended pregnancy rates** when IUDs are inserted by auxiliary nurse-midwives compared to doctors (RR 0.95, 95% CI 0.40 to 2.27, p = 0.927, low certainty evidence).
- There may be no or little difference in **referral rates**, either during IUD insertion (RR 0.80, 95% CI 0.50 to 1.29, p = 0.3731, low certainty evidence) or during follow-up after IUD insertion (RR 1.49, 95% CI 0.88 to 2.54, p = 0.1298, low certainty evidence), when IUDs are inserted by auxiliary nurse-midwives compared to doctors.
- The studies did not measure the uptake of contraceptives, complication rates at insertion or insertion failure rates.

#### Tubal ligation by midwives compared to doctors (1 RCT)

Tubal ligation performed by midwives was compared to tubal ligation performed by doctors in one RCT [23] (see Table 5 Summary of findings). This study suggests the following:

**Table 5:** **Summary of findings**

**What is the effectiveness of tubal ligation performed by midwives compared to tubal ligation performed by doctors?**						
**Patient or population:** patients with tubal ligation						
**Settings:** Hospital setting, Khon Kaen, Thailand^1^ (Dusitsin et al. [23])						
**Intervention:** Midwives performing tubal ligation						
**Comparison:** Doctors performing tubal ligation						
**Outcomes**	Illustrative comparative risks* (95% CI)		**Relative effect (95% CI)**	**No of Participants (studies)**	**Certainty of the evidence (GRADE)**	**Comments**
	Assumed risk	Corresponding risk				
	**Doctors performing tubal ligation**	**Midwives performing tubal ligation**				
Complication rates during surgery^2^	5 per 1000	11 per 1000	RR 2.12	1168	⊕⊕⊝⊝	
		(3 to 34)	(0.64 to 6.88)	(1 study)	low^3,4^	
Postoperative complications^5^	60 per 1000	70 per 1000	RR 1.16	292	⊕⊕⊝⊝	
		(29 to 161)	(0.48 to 2.66)	(1 study)	low^3,4^	
Duration of operation	The mean length of operation in the intervention groups was 6.6 minutes higher (5.58 to 7.62 minutes higher)			292	⊕⊕⊕⊝	
				(1 study)	moderate^3^	
Uptake of contraceptives^6^ - not measured	See comment	See comment	Not estimable^6^	-	See comment	
Unintended pregnancy rates^6^ - not measured	See comment	See comment	Not estimable^6^	-	See comment	

*The basis for the **assumed risk** (e.g. the median control group risk across studies) is provided in footnotes. The **corresponding risk** (and its 95% confidence interval) is based on the assumed risk in the comparison group and the **relative effect** of the intervention (and its 95% CI).

**CI:** Confidence interval; **RR:** Risk ratio;

GRADE Working Group grades of evidence.

**High certainty:** We are very confident that the true effect lies close to that of the estimate of the effect.

**Moderate certainty:** We are moderately confident in the effect estimate: The true effect is likely to be close to the estimate of the effect, but there is a possibility that it is substantially different.

**Low certainty:** Our confidence in the effect estimate is limited: The true effect may be substantially different from the estimate of the effect.

**Very low certainty:** We have very little confidence in the effect estimate: The true effect is likely to be substantially different from the estimate of effect.

^1^As the setting is not specified in the paper, we assumed that the intervention was delivered in a hospital. Midwives were recruited at Khon Khaen Maternal and Child Health Center, Thailand.

^2^Complications during surgery were reported to be due to thick abdominal fat, tubal adhesions, dextroverted uterus and inadequate sedation/analgesia.

^3^Downgraded because of differences in baseline characteristics.

^4^Downgraded because of imprecision (i.e. the confidence interval indicates both benefit and harm).

^5^Post-operative complications included mild pyrexia, respiratory infection, cystitis and wound breakdown at 5 days and 6 weeks after operation.

^6^The study did not measure uptake of contraceptives or unintended pregnancy rates.

- There may be little or no difference in **complication rates during surgery** when tubal ligation is performed by midwives compared to doctors, although the total number of events was very low (RR 2.12, 95% CI 0.64 to 6.88, p = 0.1895, low certainty evidence).
- There may be little or no difference between **postoperative complications** (such as mild pyrexia, respiratory infection, cystitis, and wound breakdown) assessed at five days and six weeks post procedure when tubal ligation is performed by midwives compared to doctors (RR 1.16, 95% CI 0.48 to 2.66, p = 0.7417, low certainty evidence).
- The **duration of a tubal ligation operation** is probably longer when performed by midwives compared to doctors. However, the time difference may not be clinically important (mean time = 18.5 minutes for midwives (SE 0.41) versus 11.9 minutes for doctors (SE 0.32), mean difference = 6.60, 95% CI 5.58 to 7.62, p < 0.0001, moderate certainty evidence).
- The study did not assess uptake of contraceptives or unintended pregnancy rates.

#### Vasectomy by medical students compared to doctors (1 RCT)

Vasectomy performed by medical students was compared to vasectomy performed by doctors in one RCT [25] (see Table 6 Summary of findings). This study suggests the following:

**Table 6:** **Summary of findings**

**What is the effectiveness of vasectomy performed by medical students compared to vasectomy performed by doctors?**						
**Patient or population:** patients with vasectomy						
**Setting:** Chulalongkorn Hospital, Bangkok, Thailand (Bunyaratavej et al. [25])						
**Intervention:** Medical students performing vasectomy						
**Comparison:** Doctors performing vasectomy						
**Outcomes**	Illustrative comparative risks* (95% CI)		**Relative effect (95% CI)**	**No of Participants (studies)**	**Certainty of the evidence (GRADE)**	**Comments**
	Assumed risk	Corresponding risk				
	**Doctors performing vasectomy**	**Medical students performing vasectomy**				
Complication rates during surgery^1^	See comment	See comment^2^	Not estimable	463 (1 study)	⊕⊕⊕⊝ moderate^3^	
Early post-operative complication rates (within 7 days)^4^	43 per 1000	33 per 1000 (13 to 85)	RR 0.78 (0.31 to 1.99)	456 (1 study)	⊕⊕⊝⊝ low^,5^	
Post-operative oligospermia rates (after 3 months)^6^	29 per 1000	76 per 1000 (25 to 225)	RR 2.59 (0.87 to 7.70)	322 (1 study)	⊕⊕⊝⊝ low^4,7^	
Unintended pregnancy rates^8^ - not measured	See comment	See comment	Not estimable^8^	-	See comment	

*The basis for the **assumed risk** (e.g. the median control group risk across studies) is provided in footnotes. The **corresponding risk** (and its 95% confidence interval) is based on the assumed risk in the comparison group and the **relative effect** of the intervention (and its 95% CI).

**CI:** Confidence interval; **RR:** Risk ratio;

GRADE Working Group grades of evidence.

**High certainty:** We are very confident that the true effect lies close to that of the estimate of the effect.

**Moderate certainty:** We are moderately confident in the effect estimate: The true effect is likely to be close to the estimate of the effect, but there is a possibility that it is substantially different.

**Low certainty:** Our confidence in the effect estimate is limited: The true effect may be substantially different from the estimate of the effect.

**Very low certainty:** We have very little confidence in the effect estimate: The true effect is likely to be substantially different from the estimate of effect.

^1^Defined as errors made in identifying and resecting the vas deferens.

^2^No complications found in either group.

^3^Downgraded because of unclear risk of contamination and blinding.

^4^Complications included bleeding (ecchymosis and/or minor hematoma(<2cms) requiring no treatment or hematoma requiring evacuation of blood clot) and infection (mild or superficial requiring no antibiotic treatment or moderately severe requiring antibiotic treatment).

^5^Downgraded because of imprecision (i.e. because of wide confidence intervals).

^6^Defined as sperm count <10,000/ml.

^7^Downgraded because of high loss to follow-up.

^8^The study did not measure unintended pregnancy rates.

- There is probably little or no difference in **complication rates during surgery** (no complications found in either group, moderate certainty evidence).
- There may be little or no difference in **early post-operative complication rates** (within seven days after the procedure) (RR 0.78, 95% CI 0.31 to 1.99, p = 0.5536, low certainty evidence) or in **oligospermia** rates three months after surgery (RR 2.59, 95% CI 0.87 to 7.70, p = 0.0865, low certainty evidence) when a vasectomy is performed by medical students compared to doctors.
- The study did not measure unintended pregnancy rates.

## Discussion

This systematic review reports the evidence on the effectiveness and safety of optimizing the delivery of a broad range of contraceptives through task shifting. The five included studies focused on three types of long-term contraceptives: insertion of IUDs, tubal ligation, and vasectomy. No eligible studies were found for injectable contraceptives or implants. In general, the studies measured safety and effectiveness in terms of contraceptive continuation, complications and failure rates during and after IUD insertion and tubal ligation or vasectomy, respectively. Overall, the results suggest that there may be little or no difference in the effectiveness and safety of contraceptive delivery compared to the performance of doctors, concerning IUD insertion by nurses and auxiliary nurse midwives with additional training, tubal ligation performed by midwives with additional training, and vasectomy performed by medical students with additional training. However, the certainty of the evidence was low or very low for many outcomes.

Few studies met the inclusion criteria of this review, and those that were selected therefore do not represent the full range of health worker cadres, contraceptive methods, and settings. No studies were found which assess the safety and effectiveness of task shifting for the delivery of contraceptive implants or injectable contraceptives. This is a major evidence gap given the importance of these methods in some settings. A recent review on safety and effectiveness of LHWs delivering injectable contraceptives identified only one study that evaluated this intervention [17]. The available evidence was of very low certainty, and the safety and effectiveness of this intervention therefore remains uncertain. In addition, we did not identify any eligible studies which assessed the impact of task shifting on contraceptive uptake rates. This is perhaps not surprising given that the study designs included in this review may not be the most commonly used or optimal designs for assessing this outcome. Reviews of other types of studies may be needed to gather data on whether implementing task shifting for the delivery of contraceptives leads to an increase in access to contraceptives at the population level.

The applicability across a range of settings of the review findings is limited for a number of reasons: the included studies are largely hospital-based and their applicability to primary care settings, especially in rural and poor areas where human resource constraints are most prevalent, is therefore unclear. Furthermore, the studies did not always sufficiently describe the level and type of training and supervision needed. The applicability of these review findings therefore needs to be assessed carefully in each setting, together with global and local evidence on the acceptability and feasibility of the interventions [26]. Important applicability considerations include, firstly, that the competencies (and titles) of specific health cadres differ across countries. It should therefore not be assumed that a cadre such as ‘auxiliary nurse-midwives’, for example, will necessarily have identical roles and responsibilities across all settings [7]. Secondly, sociocultural differences, such as levels of female literacy, may influence the success of an intervention. However, taking into consideration possible socio-cultural changes since the time period of included evidence (published between 1977 and 1995), insights gained and conclusions drawn from them may not fully apply today. Thirdly, the level of resources available for intervention implementation, including for training and supervision, may also vary between countries and this may impact on the success of implementation. Finally, government commitment to contraceptive provision as well as policy choices regarding, for instance, health workforce distribution and qualifications, may influence the provision of family planning services in LMICs [1,27,28].

Locating and identifying studies of task shifting initiatives is challenging. Although the term ‘task shifting’ may be used by authors to describe particular interventions, the term is often not indexed in electronic databases. Searching for studies involving specific health worker cadres is complicated further by the fact that the names for different health worker cadres may vary and locally-preferred alternatives or variations may be applied in different settings. A variety of terms for health worker cadres and contraceptives were used during the literature search but it is possible that some relevant studies may not have been identified. The use of a single review author during the initial screening phase of the search records may have also led to studies not being identified, as these initial judgements were not evaluated by the other authors. No language restrictions were applied, but studies published in languages such as Mandarin may have been missed in the search. Finally, it is possible that potentially eligible studies may have been excluded from the review because information about the health worker cadre and settings involved was not reported [29]. The effect of publication bias could not be assessed in this review due to the small number of studies included.

All the studies included in this review are RCTs, despite the fact that we searched for non-randomized study designs too. Other systematic reviews on task shifting have also only identified RCTs [17,30,31]. In a recent methodological study of Cochrane reviews of health systems interventions, it was noted that although Cochrane reviews of health systems interventions commonly search for non-randomized trials, the extent to which these are identified varies greatly. In particular, reviews of delivery interventions, including task shifting, have identified few non-randomized studies [32]. Collectively, this evidence may suggest that future reviews in this field should consider searching for RCTs only.

Studies assessing the safety and effectiveness of task shifting in contraceptive provision, and particularly of IUD insertion and tubal ligation, have been conducted since the 1960s [33-38]. Most have concluded that task shifting in contraceptive delivery is safe and effective. Other reviews, such as Malarcher *et al.* [13], have emphasized the safety and effectiveness of injectable contraceptives being delivered by LHWs. But a more recent review of the use of LHWs to provide injectable contraceptives concluded that it is uncertain whether the use of LHWs improves contraceptive uptake or is able to maintain patient safety and satisfaction. The review suggests, however, that where access to professional health workers is limited and where LHW programmes already exist, consideration should be given to training LHWs to administer injections with a safe injection device [17]. In their commentary on contraceptive studies, Janowitz *et al.* [8] suggest that task shifting is safe and effective for different contraceptive methods and types of health workers. Our review findings are consistent with the findings of these other reviews, at least for the limited range of contraceptives and health cadres for which studies were identified.

## Conclusions

This systematic review of task shifting for contraceptive delivery found evidence involving a limited range of interventions: the insertion of IUDs by auxiliary nurse-midwives and nurses compared to doctors; tubal ligation performed by midwives compared to doctors; and vasectomy performed by medical students compared to doctors. In general, there appears to be little or no difference in the outcomes when the contraceptive services described above are delivered by different cadres. This suggests that task shifting may be an effective and safe intervention to increase access to contraceptive delivery. However, the certainty of the evidence was generally low and the outcomes assessed differed across studies. This makes it vital that the implementation of these interventions is conducted in the context of rigorous research or targeted monitoring and evaluation [7].

**Figure 2:**
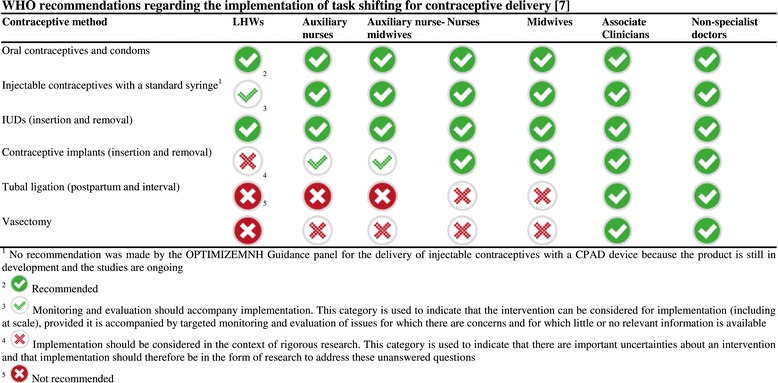
**WHO recommendations regarding the implementation of task shifting for contraceptive delivery [** 7 **].**

As noted above, the findings of this review informed the recent WHO OPTIMIZEMNH guidance [7]. Figure 2 summarizes the WHO recommendations regarding the implementation of task shifting for contraceptive delivery, including where delivery should be accompanied by further research or by monitoring and evaluation.

In addition to assessing the safety of using different cadres to deliver contraceptives, future studies should assess the extent to which task shifting impacts on contraceptive coverage within targeted populations. Future studies would also benefit from longer follow-up periods and larger study samples. Qualitative or mixed-method approaches could add value to the assessment of the acceptability and feasibility of task shifting for contraceptive interventions [27,39]. These methods could increase our understanding of how context, including sociocultural, geographical, epidemiological, health system, legal and political factors, affects the success or failure of such interventions [27]. Factors that are beyond the scope of this systematic review on safety and effectiveness but that should be explored in future studies of task shifting in contraceptive delivery include factors tied to workforce planning, education and training, regulation, support and retention. To improve comparability and replicability, these studies should also clearly describe the level of pre- and post-graduate education and the training completed by a health worker cadre prior to the implementation of task shifting. Actions taken to support health workers in their newly acquired competencies, such as revisions to practice regulations, strengthening of supervision, or provision of materials and incentives should also be reported.

## Endnotes

^a^One journal article reported two separate RCTs conducted in two different countries. The studies have been treated separately in this review and are referred to as Eren *et al*. [21] Study A and Eren *et al*. [21] Study B.

## Additional files

Additional file 1:
**EPOC study designs, definitions and exclusion criteria.**
Additional file 2:
**Search strategies.**
Additional file 3:
**Cochrane data extraction form.**
Additional file 4:
**Characteristics of the included studies.**
Additional file 5:
**Characteristics of the excluded studies.**

## Abbreviations

CBA: Controlled before-after studies
CPAD: Pre-filled injection device
EPOC: Cochrane Effective Practice and Organization of Care
GRADE: Grading of Recommendations Assessment, Development and Evaluation
IUD: Intrauterine device
ITS: Interrupted time series
LHWs: Lay health workers
LMICs: Low- and middle-income countries
NRCT: Non-randomized controlled trial
RCT: Randomized controlled trial
WHO: World Health Organization
