# Supplementary material for: Optimizing the delivery of contraceptives in low- and middle-income countries through task shifting: a systematic review of effectiveness and safety
Source: Reprod Health. 2015 Apr 1;12:27. doi: 10.1186/s12978-015-0002-2 (PMC4392779; doi:10.1186/s12978-015-0002-2)
Supplement: Additional file 1: — EPOC study designs, definitions and exclusion criteria. [file 12978_2015_2_MOESM1_ESM.docx]

**Additional file 1**

EPOC study designs, definitions and exclusion criteria (adapted from Effective Practice and Organisation of Care 2013)

| **Study design** | **Definition** | **Exclusion criteria** |
| --- | --- | --- |
| Randomized controlled trial (RCT) OR randomized trial | An experimental study which uses methods to allocate people randomly to different interventions. | *Studies with only one intervention or control site.*  EPOC recommends only including cluster randomized trials, non‑randomized cluster trials, and controlled before-after studies (CBAs) with at least two intervention sites and two control sites.  In studies with only one intervention or one control site, the intervention (or comparison) is completely confounded by study site making it difficult to attribute any observed differences to the intervention rather than to other site-specific variables. |
| Non-randomized controlled trial (NRCT) OR non‑randomized trial | An experimental study in which people are not allocated randomly to different interventions but are allocated using methods which are not random. |  |
| Controlled before-after study (CBA) | A study in which observations are made before and after the implementation of an intervention – both in a group that receives the intervention and in a control group that does not receive the intervention. |  |
| Interrupted time series study (ITS) | A study that uses observations at multiple time points before and after an intervention (the ‘interruption’). The design attempts to detect whether the intervention has had an effect significantly greater than any underlying trend over time. | *Studies that do not have a clearly defined point in time when the intervention occurred and have at least three data points before and three data points after the intervention* |

Effective Practice and Organisation of Care (EPOC) (2013): **What study designs should be included in an EPOC review and what should they be called?** *EPOC Resources for review authors.* Oslo: Norwegian Knowledge Centre for the Health Services. [<http://epocoslo.cochrane.org/epoc-specific-resources-review-authors>]
