# Supplementary material for: Optimizing the delivery of contraceptives in low- and middle-income countries through task shifting: a systematic review of effectiveness and safety
Source: Reprod Health. 2015 Apr 1;12:27. doi: 10.1186/s12978-015-0002-2 (PMC4392779; doi:10.1186/s12978-015-0002-2)
Supplement: Additional file 2: — Search strategies. [file 12978_2015_2_MOESM2_ESM.docx]

# Additional file 2: Search strategies

We searched following electronic databases:

1. PubMed (1950 - 2012 Week2), searched 17 January 2012
2. EMBASE, Ovid (1974 - 2012 Week 17), searched 2 May 2012
3. POPLINE, searched 23 February 2012
4. The Reproductive Health Library (RHL), searched 24 February 2012
5. Cochrane Central Register of Controlled Trials (CENTRAL) Trials (The Cochrane Library 2012, Issue 5), searched 4 May 2012
6. Cumulative Index of Nursing and Allied Health (CINAHL) (1985-2011), searched 08 May 2012
7. The Global Health Library (GHL), searched 25 May 2012, including WHOLIS Regional databases (African Index Medicus (AFRO), Index Medicus for South-East Asia (SEARO), Western Pacific Regional Index Medicus (WPRO), Literatura Latinoamericana y del Caribe en Ciencias de la Salud (LILACS), Index Medicus for WHO Eastern Mediterranean IMEMR (EMRO)
8. **PubMed Search Strategy**

**Concept 1: contraception**

Sterilization, Tubal [MesH] OR Tubal Sterilization* [tw] OR Aldridge Procedure [TW] OR Cooke Method [TW] OR Cornual Coagulation [tw] OR Fimbriectom* [TW] OR Irving Method [TW] OR Tubal Rings [TW] OR Tubal Ring [TW] OR Tubal Ligations [TW] OR Tubal Ligation [TW] OR Madlener Method [TW] OR Pomeroy Method [TW] OR Pomeroy Sterilization [TW] OR Tubal Excision* [TW] OR Tubal Occlusion OR Tubal Occlusions [TW] OR Uchida Method [TW] OR Kroener Fimbriectomy [TW] OR Kroener Method [TW]

Vasectomy [Mesh] OR Vasectomies [TW] OR vasectomy [TW] OR Vas Ligation [TW] OR Vas Ligations [TW] OR Vas Occlusion* [TW] OR Intravasal Thread [TW] OR Intravasal Thread* [TW]

Intrauterine Devices [MeSH] OR Intrauterine Devices [tw] OR Intrauterine Device [tw] OR Contraceptive IUD [tw] OR Contraceptive IUDs [tw] OR Intrauterine Contraceptive Device* [tw] OR Unmedicated IUDs [tw] OR Unmedicated IUD [tw]

Drug implants [MeSH] OR Drug implants [tw] OR drug implant [tw] OR Drug Pellets [tw] OR Levonorgestrel [MeSH] OR Norethindrone [MeSH] OR contraceptive implants [tw] OR progestogen only contraceptives OR contraceptive implant [tw] OR progestogen implants [tw] OR etonogestrel implants [tw] OR Implanon [tw] OR Subdermal contraceptive implant* [tw] OR Norplant [tw] OR Jadelle [tw] OR Sino-implant[tw] Nexplanon [tw] OR Norprogesterones [tw]

Condoms [MeSH] OR Condom* [tw] OR Condom Manufacture [tw]

Oral Contraceptives [MeSH] OR Oral Contraceptives [tw] OR Oral Contraceptive [tw]

Contraception [MeSH] OR Contraception [tw] OR Contraceptive Methods [tw] OR Contraceptive Method [tw] OR Inhibition of Fertilization [tw] OR Fertilization Inhibition [tw] OR Birth Control [tw] OR Fertility Control [tw] OR Female Contraception [tw] OR male Contraception [tw] OR contracept* [tw] OR anticoncept* [tw]

Contraceptive Agents [MeSH] OR Contraceptive Agents [tw] OR contraceptives [tw]

Contraception Behavior [MeSH] OR Contraception Behaviors [tw] OR Contraception Behaviour [tw] OR Contraception Behaviors [tw] OR Contraceptive Behav* [tw] OR Contraceptive Usage [tw] OR Contraceptive Method Switching [tw]

“injections”[MeSH] OR “injections”[tiab] OR “injectable”[tiab] OR “algestone acetophenide” [tiab] OR DMPA [tiab] OR deladroxate [tiab] OR “dihydroxyprogesterone acetophenide” [tiab] OR estradiol cypionate [tiab] OR estradiol 17 beta-cypionate [tiab] OR estradiol valerate [tiab] OR medroxyprogesterone acetate [tiab] OR medroxyprogesterone 17-acetate [tiab] OR medroxyprogesterone acetate-17 [tiab] OR 17-medroxyprogesterone acetate [tiab] OR mpa [tiab] OR NET-EN [tiab] OR NET-ENT [tiab] OR NET-OEN [tiab] OR noresterat [tiab] OR norethindrone enanthate [tiab] OR norethindrone oenanthate [tiab] OR norethisterone enanthate [tiab] OR norethisterone oenanthate [tiab] OR Depoprovera OR Depo-provera OR Curretab [tiab] OR  Cycrin [tiab] OR “Depo-Provera” [tiab] OR “Depo Provera” [tiab] OR DepoProvera[tiab] OR Farlutal [tiab] OR Perlutex [tiab] OR Provera [tiab] OR Veramix [tiab] OR Clinovir [tiab] OR Gestapuran [tiab]

**Concept 2: health cadres**

Community Health Aides [MeSH] OR community health aides [tw] OR Village Health Worker [tw] OR Village Health Workers [tw] OR Family Planning Personnel [tw] OR Family Planning Personnel Characteristics [tw] OR Barefoot Doctors [tw] OR Barefoot Doctor [tw] OR Community Workers [tw] OR Community Worker [tw]

Allied health personnel [MeSH] OR Allied health personnel [tw] OR Paramedics [tw] OR Paramedic [tw] OR Paramedical Personnel [tw] OR Population Program Specialists [tw] OR Population Program Specialist [tw]

Home Health Aides [MeSH] OR Home Health Aides [tw] OR Home Health Aide [tw] OR Homemaker-Home Health Aides [tw] OR Homemaker Home Health Aides [tw] OR Homemaker-Home Health Aide [tw] OR Home Care Aides [tw] OR Home Care Aide [tw]

Voluntary Workers [MeSH] OR Voluntary Workers [tw] OR Voluntary Worker [tw] OR Volunteer Workers [tw] OR Volunteerism [tw] OR Untrained Personnel [tw]

Home Nursing [MeSH] OR Home Nursing [tw] OR Nonprofessional Home Care [tw]

Peer Group [MeSH] OR Social Support [MeSH]

Lay health worker [tw] OR lay health workers [tw] OR lay health volunteer* [tw] OR Community health worker* [tw] OR treatment supporter* [tw] OR birth attendants [tw] OR Shasthyo Sebika [tw] OR Community Nutrition Worker [tw] OR Village Health Workers [tw] OR Nutrition Volunteers [tw] OR Community Health Agents [tw] OR Agente comunitário de saúde [tw] OR Visitador* [tw] OR Women Group Leader* [tw] OR Maternal Health Worker* [tw] OR Maternal Child Health Worker* [tw] OR Voluntary Malaria Worker* [tw] OR Raedat [tw] OR Mental Health Worker* [tw] OR Postnatal Support Worker* [tw] OR Village Malaria Worker* [tw] OR Village Health Promoter* [tw] OR Rural Health Worker* [tw] OR Accompagnateur* [tw] OR Saksham Sahaya [tw] OR Anganwadi worker* [tw] OR maternal and Child Health Promotion Worker* [tw] OR Community based Workers [tw] OR Community Health Volunteer* [tw] OR Village Health Guide* [tw] OR Behvarz [tw] OR Female Community Health Volunteer* [tw] OR Brigadistas [tw] OR Lady Health Worker* [tw] OR Agente Comunitario de Salud [tw] OR Nutrition Worker* [tw] OR Community Drug Distributor* [tw] OR Lay Health Visitor* [tw] OR Community Volunteer* [tw] OR Community Health Advocate* [tw] OR Community Health Aide* [tw] OR Promotora* [tw] OR Lay volunteer* [tw] OR lay worker [tw] OR lay visitor [tw] OR lay attendant [tw] OR lay aide [tw] OR lay aides [tw] OR lay support* [tw] OR lay person* [tw] OR lay helper [tw] OR lay caregiver* [tw] OR lay consultant [tw] OR lay assistant [tw] OR lay staff [tw] OR lay visit* [tw] OR lay midwife [tw] OR lay midwives [tw] OR volunteer worker [tw]

Paraprofessional* [tw] OR paramedic [tw] OR paramedics [tw] OR paramedical worker* [tw] OR paramedical personnel [tw] OR allied health personnel [tw] OR allied health worker* [tw] OR support worker* [tw] OR home health aide*[tw]

Trained volunteer* [tw] OR trained health worker* [tw] OR trained mother* [tw] OR trained healthcare worker* [tw] OR trained health care worker*[tw]

Birth attendan* [tw] OR trained birth attendan* [tw] OR labour attendan* [tw] OR labour assistant* [tw] OR labor attendant* [tw] OR labor assistant* [tw] OR doula* [tw] OR douladural* [tw] OR monitrice [tw] OR childbirth assistant* [tw] OR childbirth attendant* [tw] OR traditional birth attendant* [tw] OR skilled birth attendant* [tw] OR traditional midwives [tw] OR dayas [tw] or Dai [tw]

Peer volunteer* [tw] OR peer counsel* [tw] OR peer support [tw] OR peer intervention [tw]

OR Church based intervention* [tw] or church based program*[tw] or church based counsel*[tw] OR Linkworker* [tw] OR link worker [tw]

Nurses' Aides [MeSH] OR nurses' aides [tw] OR nurses aide [tw] OR Nurses Aides [tw] OR Nurses' Aide [tw] OR nurse aide* [tw] OR Nursing Auxiliaries [tw] OR Nursing Auxiliary [tw] OR Auxiliary nurse* [tw] OR auxiliary nurse midwife [tw] OR auxiliary nurse midwives [tw] OR auxiliary midwife [tw] OR auxiliary midwives [tw] OR nurse assistant [tw]

Nurse [MeSH] OR nurse* [tw] OR Nursing Personnel [tw] OR Registered nurse* [tw] OR nurse practitioner* [tw] OR clinical nurse specialist* [tw] OR advanced practice nurse* [tw] OR practice nurse* [tw] OR licensed nurse* [tw] OR diploma nurse* [tw] OR BS nurse* [tw] OR nurse clinician* [tw]

Midwifery [MeSH] OR midwifery [tw] OR midwives [tw] OR Registered midwife [tw] OR midwife [tw] OR community midwife [tw]

Non-physician clinician* [tw] OR non-professional clinician [tw] OR Clinical officer [tw] OR medical assistant* [tw] OR physician assistant* [tw] OR surgical technician* [tw] OR non-professional clinician [tw] OR non-clinician medical practitioners [tw] OR non-clinician* OR Non-specialist doctors [tw]

**Concept 3: EPOC methodology**

#1. randomized controlled trial[pt]

#2. random*[tiab]

#3. intervention*[tiab]

#4. control[tiab] or controlled[tiab]

#5. evaluat*[tiab]

#6. #1 or #2 or #3 or #4 or #5

#7. Animals[MeSH]

#8. Humans[MeSH]

#9. #7 not (#7 and #8)

#10. #6 not #9

### Embase

1. ('lay volunteer' or 'lay volunteers').mp.

2. (paraprofessional* or paramedic or paramedics or 'paramedical worker' or 'paramedical workers' or 'paramedical personnel' or 'allied health personnel' or 'allied health worker' or 'allied health workers' or 'support worker' or 'support workers' or 'home health aide' or 'home health aides').

3. (trained adj3 (volunteer* or 'health worker' or 'health workers' or mother*)).ab,ti.

4. ((lay or voluntary or volunteer* or untrained or unlicensed or nonprofessional* or 'non professional' or 'non professionals') adj5 (worker* or visitor* or attendant* or aide or aides or support* or person* or helper* or carer* or caregiver* or 'care giver' or 'care givers' or consultant* or assistant* or staff or visit* or midwife or midwives)).ab,ti.

5. ((community or village*) adj3 ('health worker' or 'health workers' or 'health care worker' or 'health care workers' or 'healthcare worker' or 'healthcare workers')).ab,ti.

6. ((birth or childbirth or labor or labour) adj1 (attendant* or assistant*)).ab,ti.

7. (doula* or douladural*).ab,ti.

8. monitrice*.ab,ti.

9. (peer adj1 (volunteer* or counsel* or support or intervention*)).ab,ti.

10. (community adj3 (volunteer* or aide or aides or support)).ab,ti.

11. ('church based' adj3 (intervention* or program* or counsel*)).ab,ti.

14. (linkworker* or 'link worker' or 'link workers')

15. ('barefoot doctor' or 'barefoot doctors').ti,ab.

16. outreach*.ti,ab.

17. (home adj1 (care or aide or aides or nursing or support or intervention* or treatment* or visit*)).ab,ti.

18. ((care or aide or aides or nursing or support or intervention* or treatment* or visit*) adj3 (lay or volunteer* or voluntary)).ab,ti.

19. 17 and 18

20. exp voluntary worker/

21. exp paramedical personnel/

22. exp health auxiliary/

23. exp peer group/

24. exp doula/

25. exp paramedical personnel/ or "allied health personnel".mp. or "para medical personnel".mp. or paramedical assistant.mp. or paramedical manpower.mp. or paramedical professional.mp. or paramedical staff.mp. or doula.mp. or doulas.mp. or health visitor*.mp. or "allied health profession".mp. or "operating room personnel".mp. or "health care personnel".mp. or "operating room".mp. or "operating department assistant".mp. or "operating department practitioner".mp. or "operating room nursing".mp. or "operating room technicians".mp. or "surgical staff".mp. or "surgical technician".mp. or "surgical technologist".mp. (mp=title, abstract, subject headings, heading word, drug trade name, original title, device manufacturer, drug manufacturer, device trade name, keyword)

26. (pediatric assistants*or physicians' assistant* or physician assistant*).ti,ab.

27. exp health auxiliary/ or barefoot doctor?.mp. or community health aide?.mp. or community health worker.mp. or community health worker?.mp. or medical auxiliary*.mp. or health auxiliaries.mp. (mp=title, abstract, subject headings, heading word, drug trade name, original title, device manufacturer, drug manufacturer, device trade name, keyword)

28. (Nurse or nurses or community health nurse or nurse clinicians or nurse,community health or public health nurse).mp. or exp advanced practice nurse/ or clinical nurse specialist.mp. or exp nurse practitioner/ or nurse midwives.mp. or family nurse practitioner.mp. or registered nurse.mp. (mp=title, abstract, subject headings, heading word, drug trade name, original title, device manufacturer, drug manufacturer, device trade name, keyword)

29. (midwife or midwifery or midwives or nurse midwife or nurse midwives).mp. (mp=title, abstract, subject headings, heading word, drug trade name, original title, device manufacturer, drug manufacturer, device trade name, keyword)

31. (auxiliary nurse or nurse auxiliar* or auxiliary nurses or anaesthesist nurse assistant or anesthetist nurse assistant or nursing assistance or nursing assistant or nurse's aide or nurses' aides or nursing aid or nursing aide or orderlies).ti,ab.

32. (Nurse or nurses or "community health nurse" or "nurse clinicians" or "community health nurse" or "public health nurse" or "advanced practice nurse" or "clinical nurse specialist" or "nurse practitioner" or "nurse midwives" or "family nurse practitioner" or "registered nurse").mp.

33. (barefoot doctor? or community health aide? or community health worker or community health worker? or medical auxiliary* or health auxiliaries).mp. or exp health auxiliary/ (mp=title, abstract, subject headings, heading word, drug trade name, original title, device manufacturer, drug manufacturer, device trade name, keyword)

34. 1 or 2 or 3 or 4 or 5 or 6 or 7 or 8 or 9 or 10 or 11 or 12 or 13 or 14 or 15 or 16 or 19 or 20 or 22 or 23 or 24 or 25 or 26 or 30 or 31 or 32 or 33

35. exp uterine tube sterilization/ or exp female sterilization/ or exp uterine tube surgery/ or exp salpingectomy/ or exp uterine tube coagulation/ or exp uterine tube ligation/ or uterine tube sterilization.ti,ab. or female sterilization.ti,ab. or uterine tube surgery.ti,ab. or salpingectomy.ti,ab. or uterine tube coalgulation.ti,ab. or uterine tube ligationt.ti,ab. or uterine tube sterilization.ti,ab.

36. exp vasectomy/ or vasectomy.ti,ab. or bilateral vasectomy.ti,ab. or spermatic cord resection.ti,ab.

37. (intrauterine device? or intrauterine contraceptive device? or (IUD* or IUCD* or IUS*) or intrauterine W device or intrauterine w contracept* or intrauterine system).mp. (mp=title, abstract, subject headings, heading word, drug trade name, original title, device manufacturer, drug manufacturer, device trade name, keyword)

38. (contracepti$ adj implant$).ti,ab. or LEVONORGESTREL/ or NORGESTREL/ or Norplant$.ti,ab. or Uniplant.ti,ab. or (keto adj desogestrel).ti,ab. or levonorgestrel.ti,ab. or norgestrel.ti,ab. or etonorgestrel.ti,ab. or implanon.ti,ab. or jadelle.ti,ab. or nestorone.ti,ab. or elcometrine.ti,ab. or normegestrol.ti,ab. or (subdermal adj implant$).ti,ab.

39. exp drug implant/ or exp drug implantation/ or (drug implant*.mp. adj5 exp contraceptive agent/) or contraceptive agents.mp. (mp=title, abstract, subject headings, heading word, drug trade name, original title, device manufacturer, drug manufacturer, device trade name, keyword)

40. 38 or 39

41. exp Condom/ or condome.ti,ab. or condoms.ti,ab. or exp contraceptive devices, male/ or male contraceptive devices.mp.

42. exp oral contraceptive agent/ or oral contraceptives.ti,ab. or oral contraception.ti,ab.

43. exp injectable contraceptive agent/ or exp algestone acetofenide plus estradiol enanthate/ or exp chorionic gonadotropin vaccine/ or exp contraceptive vaccine/ or exp ectylurea plus ethoxzolamide plus medroxyprogesterone acetate/ or exp estradiol cypionate plus medroxyprogesterone acetate/ or exp estradiol enanthate/ or exp estradiol valerate plus norethisterone enantate/ or exp medroxyprogesterone acetate/ or exp norethisterone enantate/

(algestone acetofenide plus estradiol enanthate or chorionic gonadotropin vaccine or contraceptive vaccine or ectylurea plus ethoxzolamide plus medroxyprogesterone acetate or estradiol cypionate plus medroxyprogesterone acetate or estradiol enanthate or estradiol valerate plus norethisterone enantate or medroxyprogesterone acetate or norethisterone enantate or Curretab OR  Cycrin OR “Depo-Provera” OR “Depo Provera” OR DepoProvera OR Farlutal OR Perlutex OR Provera OR Veramix OR Clinovir OR Gestapuran).mp.

44. (injectable contraceptive agent or antifertility injection or injectable contraceptive?).mp. or (inject* adj contracept*).mp.

45. 43 or 44

46. 35 or 36 or 37 or 40 or 41 or 42 or 45

47. 34 and 46

48. Developing Country.sh.

49. (Africa or Asia or Caribbean or West Indies or South America or Latin America or Central America).hw,ti,ab,cp.

50. (Afghanistan or Albania or Algeria or Angola or Antigua or Barbuda or Argentina or Armenia or Armenian or Aruba or Azerbaijan or Bahrain or Bangladesh or Barbados or Benin or Byelarus or Byelorussian or Belarus or Belorussian or Belorussia or Belize or Bhutan or Bolivia or Bosnia or Herzegovina or Hercegovina or Botswana or Brazil or Bulgaria or Burkina Faso or Burkina Fasso or Upper Volta or Burundi or Urundi or Cambodia or Khmer Republic or Kampuchea or Cameroon or Cameroons or Cameron or Camerons or Cape Verde or Central African Republic or Chad or Chile or China or Colombia or Comoros or Comoro Islands or Comores or Mayotte or Congo or Zaire or Costa Rica or Cote d'Ivoire or Ivory Coast or Croatia or Cuba or Cyprus or Czechoslovakia or Czech Republic or Slovakia or Slovak Republic or Djibouti or French Somaliland or Dominica or Dominican Republic or East Timor or East Timur or Timor Leste or Ecuador or Egypt or United Arab Republic or El Salvador or Eritrea or Estonia or Ethiopia or Fiji or Gabon or Gabonese Republic or Gambia or Gaza or Georgia Republic or Georgian Republic or Ghana or Gold Coast or Greece or Grenada or Guatemala or Guinea or Guam or Guiana or Guyana or Haiti or Honduras or Hungary or India or Maldives or Indonesia or Iran or Iraq or Isle of Man or Jamaica or Jordan or Kazakhstan or Kazakh or Kenya or Kiribati or Korea or Kosovo or Kyrgyzstan or Kirghizia or Kyrgyz Republic or Kirghiz or Kirgizstan or Lao PDR or Laos or Latvia or Lebanon or Lesotho or Basutoland or Liberia or Libya or Lithuania or Macedonia or Madagascar or Malagasy Republic or Malaysia or Malaya or Malay or Sabah or Sarawak or Malawi or Nyasaland or Mali or Malta or Marshall Islands or Mauritania or Mauritius or Agalega Islands or Mexico or Micronesia or Middle East or Moldova or Moldovia or Moldovian or Mongolia or Montenegro or Morocco or Ifni or Mozambique or Myanmar or Myanma or Burma or Namibia or Nepal or Netherlands Antilles or New Caledonia or Nicaragua or Niger or Nigeria or Northern Mariana Islands or Oman or Muscat or Pakistan or Palau or Palestine or Panama or Paraguay or Peru or Philippines or Philipines or Phillipines or Phillippines or Poland or Portugal or Puerto Rico or Romania or Rumania or Roumania or Russia or Russian or Rwanda or Ruanda or Saint Kitts or St Kitts or Nevis or Saint Lucia or St Lucia or Saint Vincent or St Vincent or Grenadines or Samoa or Samoan Islands or Navigator Island or Navigator Islands or Sao Tome or Saudi Arabia or Senegal or Serbia or Montenegro or Seychelles or Sierra Leone or Slovenia or Sri Lanka or Ceylon or Solomon Islands or Somalia or Sudan or Suriname or Surinam or Swaziland or Syria or Tajikistan or Tadzhikistan or Tadjikistan or Tadzhik or Tanzania or Thailand or Togo or Togolese Republic or Tonga or Trinidad or Tobago or Tunisia or Turkey or Turkmenistan or Turkmen or Uganda or Ukraine or Uruguay or USSR or Soviet Union or Union of Soviet Socialist Republics or Uzbekistan or Uzbek or Vanuatu or New Hebrides or Venezuela or Vietnam or Viet Nam or West Bank or Yemen or Yugoslavia or Zambia or Zimbabwe or Rhodesia).hw,ti,ab,cp.

51. ((developing or less* developed or under developed or underdeveloped or middle income or low* income or underserved or under served or deprived or poor*) adj (countr* or nation? or population? or world)).ti,ab.

52. ((developing or less* developed or under developed or underdeveloped or middle income or low* income) adj (economy or economies)).ti,ab.

53. (low* adj (gdp or gnp or gross domestic or gross national)).ti,ab.

54. (low adj3 middle adj3 countr*).ti,ab.

55. (lmic or lmics or third world or lami countr*).ti,ab.

56. transitional countr*.ti,ab.

57. 48 or 49 or 50 or 51 or 52 or 53 or 54 or 55 or 56

58. 47 and 57

1. **K4Health/POPLINE**

Keywords/Title:

(UNTRAINED PERSONNEL / BAREFOOT DOCTORS / COMMUNITY-BASED DISTRIBUTION WORKERS / COMMUNITY WORKERS / Neighborhood Workers / ="Community Health Services" / VOLUNTEERS AND VOLUNTARISM / "Human Volunteers" / ="Nurse Midwives" / TRADITIONAL BIRTH ATTENDANTS / ="Nurses and Nursing" / ="Midwives and Midwifery" / ="Peer Educators" / ="Physicians"/ ="Family Planning Instructors" / FAMILY PLANNING PERSONNEL / ="Untrained Personnel" / ="Paramedical Personnel" / Auxiliary Health Workers / ="Nonclinical Distribution") & (CONTRACEPTIVE IMPLANTS / Implants / Norplant / IUD / IUCD/ Intrauterine Device / IUD, COPPER RELEASING / IUD, HORMONE RELEASING / IUD, UNMEDICATED / CONDOMS / Nirodh / Preethi / CONTRACEPTIVE PATCH, COMBINED / ORAL CONTRACEPTIVES / ORAL CONTRACEPTIVES, COMBINED / ORAL CONTRACEPTIVES, LOW-DOSE / VAGINAL RINGS / SEXUAL STERILIZATION / Sterilization, Sexual / FEMALE STERILIZATION / TUBAL LIGATION / CORNUAL COAGULATION / TUBAL EXCISION / MALE STERILIZATION / VAS LIGATION / VAS OCCLUSION / VASECTOMY)

### Cochrane Central Register of Controlled Trials (CENTRAL)

#1 [MeSH descriptor **Contraceptive Agents** explode all trees](http://onlinelibrary.wiley.com/o/cochrane/searchHistory?mode=runquery&qnum=1) 1904 [edit](http://onlinelibrary.wiley.com/search-web/cochrane/searchHistory?mode=editquery&qnum=1&searchKey=3be65063-a79e-4d5b-a2fb-3216df124862) [delete](http://onlinelibrary.wiley.com/search-web/cochrane/searchHistory?mode=deletequery&qnum=1&uuid=3be65063-a79e-4d5b-a2fb-3216df124862&searchKey=3be65063-a79e-4d5b-a2fb-3216df124862)

#2 [IUD* OR intrauterine device* or IUCD* OR intrauterine contraceptive device*](http://onlinelibrary.wiley.com/o/cochrane/searchHistory?mode=runquery&qnum=2) 936 [edit](JavaScript:doPopup('/search-web/cochrane/searchHistory?mode=editquery&qnum=2&searchKey=3be65063-a79e-4d5b-a2fb-3216df124862',%20400)) [delete](http://onlinelibrary.wiley.com/search-web/cochrane/searchHistory?mode=deletequery&qnum=2&uuid=3be65063-a79e-4d5b-a2fb-3216df124862&searchKey=3be65063-a79e-4d5b-a2fb-3216df124862)

#3 [Vasectomy OR vasectomies or "male sterilization"](http://onlinelibrary.wiley.com/o/cochrane/searchHistory?mode=runquery&qnum=3) 120 [edit](JavaScript:doPopup('/search-web/cochrane/searchHistory?mode=editquery&qnum=3&searchKey=3be65063-a79e-4d5b-a2fb-3216df124862',%20400)) [delete](http://onlinelibrary.wiley.com/search-web/cochrane/searchHistory?mode=deletequery&qnum=3&uuid=3be65063-a79e-4d5b-a2fb-3216df124862&searchKey=3be65063-a79e-4d5b-a2fb-3216df124862)

#4 [Tubal Ligation OR tubal occlusion OR tubal sterilization OR "female sterilization"](http://onlinelibrary.wiley.com/o/cochrane/searchHistory?mode=runquery&qnum=4) 472 [edit](JavaScript:doPopup('/search-web/cochrane/searchHistory?mode=editquery&qnum=4&searchKey=3be65063-a79e-4d5b-a2fb-3216df124862',%20400)) [delete](http://onlinelibrary.wiley.com/search-web/cochrane/searchHistory?mode=deletequery&qnum=4&uuid=3be65063-a79e-4d5b-a2fb-3216df124862&searchKey=3be65063-a79e-4d5b-a2fb-3216df124862)

#5 [MeSH descriptor **Sterilization, Reproductive** explode all trees](http://onlinelibrary.wiley.com/o/cochrane/searchHistory?mode=runquery&qnum=5) 317 [edit](http://onlinelibrary.wiley.com/search-web/cochrane/searchHistory?mode=editquery&qnum=5&searchKey=3be65063-a79e-4d5b-a2fb-3216df124862) [delete](http://onlinelibrary.wiley.com/search-web/cochrane/searchHistory?mode=deletequery&qnum=5&uuid=3be65063-a79e-4d5b-a2fb-3216df124862&searchKey=3be65063-a79e-4d5b-a2fb-3216df124862)

#6 [MeSH descriptor **Contraception, Barrier** explode all trees](http://onlinelibrary.wiley.com/o/cochrane/searchHistory?mode=runquery&qnum=6) 6 [edit](http://onlinelibrary.wiley.com/search-web/cochrane/searchHistory?mode=editquery&qnum=6&searchKey=3be65063-a79e-4d5b-a2fb-3216df124862) [delete](http://onlinelibrary.wiley.com/search-web/cochrane/searchHistory?mode=deletequery&qnum=6&uuid=3be65063-a79e-4d5b-a2fb-3216df124862&searchKey=3be65063-a79e-4d5b-a2fb-3216df124862)

#7 [condom OR condoms](http://onlinelibrary.wiley.com/o/cochrane/searchHistory?mode=runquery&qnum=7) 1092 [edit](JavaScript:doPopup('/search-web/cochrane/searchHistory?mode=editquery&qnum=7&searchKey=3be65063-a79e-4d5b-a2fb-3216df124862',%20400)) [delete](http://onlinelibrary.wiley.com/search-web/cochrane/searchHistory?mode=deletequery&qnum=7&uuid=3be65063-a79e-4d5b-a2fb-3216df124862&searchKey=3be65063-a79e-4d5b-a2fb-3216df124862)

#8 [MeSH descriptor **Injections** explode all trees](http://onlinelibrary.wiley.com/o/cochrane/searchHistory?mode=runquery&qnum=8) 16910 [edit](http://onlinelibrary.wiley.com/search-web/cochrane/searchHistory?mode=editquery&qnum=8&searchKey=3be65063-a79e-4d5b-a2fb-3216df124862) [delete](http://onlinelibrary.wiley.com/search-web/cochrane/searchHistory?mode=deletequery&qnum=8&uuid=3be65063-a79e-4d5b-a2fb-3216df124862&searchKey=3be65063-a79e-4d5b-a2fb-3216df124862)

#9 [MeSH descriptor **Contraception** explode all trees](http://onlinelibrary.wiley.com/o/cochrane/searchHistory?mode=runquery&qnum=9) 285 [edit](http://onlinelibrary.wiley.com/search-web/cochrane/searchHistory?mode=editquery&qnum=9&searchKey=3be65063-a79e-4d5b-a2fb-3216df124862) [delete](http://onlinelibrary.wiley.com/search-web/cochrane/searchHistory?mode=deletequery&qnum=9&uuid=3be65063-a79e-4d5b-a2fb-3216df124862&searchKey=3be65063-a79e-4d5b-a2fb-3216df124862)

#10 [(#8 AND #9)](http://onlinelibrary.wiley.com/o/cochrane/searchHistory?mode=runquery&qnum=10) 12 [edit](JavaScript:doPopup('/search-web/cochrane/searchHistory?mode=editquery&qnum=10&searchKey=3be65063-a79e-4d5b-a2fb-3216df124862',%20400)) [delete](http://onlinelibrary.wiley.com/search-web/cochrane/searchHistory?mode=deletequery&qnum=10&uuid=3be65063-a79e-4d5b-a2fb-3216df124862&searchKey=3be65063-a79e-4d5b-a2fb-3216df124862)

#11 [dihydroxyprogesterone acetophenide OR algestone acetophenide OR medroxyprogesterone 17-acetate OR norethisterone oenanthate OR norethindrone OR (inject* NEAR/5 contracept*) OR (injectable AND contracept*) or DMPA or DepoProvera or Depo-Provera or Depo Provera](http://onlinelibrary.wiley.com/o/cochrane/searchHistory?mode=runquery&qnum=11) 1053 [edit](JavaScript:doPopup('/search-web/cochrane/searchHistory?mode=editquery&qnum=11&searchKey=3be65063-a79e-4d5b-a2fb-3216df124862',%20400)) [delete](http://onlinelibrary.wiley.com/search-web/cochrane/searchHistory?mode=deletequery&qnum=11&uuid=3be65063-a79e-4d5b-a2fb-3216df124862&searchKey=3be65063-a79e-4d5b-a2fb-3216df124862)

#12 [(#10 OR #11)](http://onlinelibrary.wiley.com/o/cochrane/searchHistory?mode=runquery&qnum=12) 1057 [edit](JavaScript:doPopup('/search-web/cochrane/searchHistory?mode=editquery&qnum=12&searchKey=3be65063-a79e-4d5b-a2fb-3216df124862',%20400)) [delete](http://onlinelibrary.wiley.com/search-web/cochrane/searchHistory?mode=deletequery&qnum=12&uuid=3be65063-a79e-4d5b-a2fb-3216df124862&searchKey=3be65063-a79e-4d5b-a2fb-3216df124862)

#13 [MeSH descriptor **Norpregnenes** explode all trees](http://onlinelibrary.wiley.com/o/cochrane/searchHistory?mode=runquery&qnum=13) 1957 [edit](http://onlinelibrary.wiley.com/search-web/cochrane/searchHistory?mode=editquery&qnum=13&searchKey=3be65063-a79e-4d5b-a2fb-3216df124862) [delete](http://onlinelibrary.wiley.com/search-web/cochrane/searchHistory?mode=deletequery&qnum=13&uuid=3be65063-a79e-4d5b-a2fb-3216df124862&searchKey=3be65063-a79e-4d5b-a2fb-3216df124862)

#14 [(contracepti* near implant*) OR (exp NORGESTREL/) OR (LEVONORGESTREL/) or norplant* or uniplant or (keto near desogestrel) or levonorgestrel or norgestrel or etonorgestrel or implanon or jadelle or nestorone or elcometrine or normegestrol](http://onlinelibrary.wiley.com/o/cochrane/searchHistory?mode=runquery&qnum=14) 1243 [edit](JavaScript:doPopup('/search-web/cochrane/searchHistory?mode=editquery&qnum=14&searchKey=3be65063-a79e-4d5b-a2fb-3216df124862',%20400)) [delete](http://onlinelibrary.wiley.com/search-web/cochrane/searchHistory?mode=deletequery&qnum=14&uuid=3be65063-a79e-4d5b-a2fb-3216df124862&searchKey=3be65063-a79e-4d5b-a2fb-3216df124862)

#15 [MeSH descriptor **Contraceptives, Oral** explode all trees](http://onlinelibrary.wiley.com/o/cochrane/searchHistory?mode=runquery&qnum=15) 1343 [edit](http://onlinelibrary.wiley.com/search-web/cochrane/searchHistory?mode=editquery&qnum=15&searchKey=3be65063-a79e-4d5b-a2fb-3216df124862) [delete](http://onlinelibrary.wiley.com/search-web/cochrane/searchHistory?mode=deletequery&qnum=15&uuid=3be65063-a79e-4d5b-a2fb-3216df124862&searchKey=3be65063-a79e-4d5b-a2fb-3216df124862)

#16 ["Oral contraceptives" or "OCs" OR "oral contraception"](http://onlinelibrary.wiley.com/o/cochrane/searchHistory?mode=runquery&qnum=16) 1443 [edit](JavaScript:doPopup('/search-web/cochrane/searchHistory?mode=editquery&qnum=16&searchKey=3be65063-a79e-4d5b-a2fb-3216df124862',%20400)) [delete](http://onlinelibrary.wiley.com/search-web/cochrane/searchHistory?mode=deletequery&qnum=16&uuid=3be65063-a79e-4d5b-a2fb-3216df124862&searchKey=3be65063-a79e-4d5b-a2fb-3216df124862)

#17 [(#15 OR #16)](http://onlinelibrary.wiley.com/o/cochrane/searchHistory?mode=runquery&qnum=17) 2055 [edit](JavaScript:doPopup('/search-web/cochrane/searchHistory?mode=editquery&qnum=17&searchKey=3be65063-a79e-4d5b-a2fb-3216df124862',%20400)) [delete](http://onlinelibrary.wiley.com/search-web/cochrane/searchHistory?mode=deletequery&qnum=17&uuid=3be65063-a79e-4d5b-a2fb-3216df124862&searchKey=3be65063-a79e-4d5b-a2fb-3216df124862)

#18 [(#1 OR #2 OR #3 OR #4 OR #5 OR #6 OR #7 OR #12 OR #13 OR #14 OR #17)](http://onlinelibrary.wiley.com/o/cochrane/searchHistory?mode=runquery&qnum=18) 5995 [edit](JavaScript:doPopup('/search-web/cochrane/searchHistory?mode=editquery&qnum=18&searchKey=3be65063-a79e-4d5b-a2fb-3216df124862',%20400)) [delete](http://onlinelibrary.wiley.com/search-web/cochrane/searchHistory?mode=deletequery&qnum=18&uuid=3be65063-a79e-4d5b-a2fb-3216df124862&searchKey=3be65063-a79e-4d5b-a2fb-3216df124862)

#19 [Nurses Or Nurse Administrators OR Nurse Anesthetists OR Nurse Clinicians OR Nurse Midwives OR Nurse Practitioners OR Male Nurses in Trials](http://onlinelibrary.wiley.com/o/cochrane/searchHistory?mode=runquery&qnum=19) 20986 [edit](JavaScript:doPopup('/search-web/cochrane/searchHistory?mode=editquery&qnum=19&searchKey=3be65063-a79e-4d5b-a2fb-3216df124862',%20400)) [delete](http://onlinelibrary.wiley.com/search-web/cochrane/searchHistory?mode=deletequery&qnum=19&uuid=3be65063-a79e-4d5b-a2fb-3216df124862&searchKey=3be65063-a79e-4d5b-a2fb-3216df124862)

#20 [Nurses' Aides OR nurse aides or auxiliary nurs* OR (nurse* NEAR/3 assistan*)](http://onlinelibrary.wiley.com/o/cochrane/searchHistory?mode=runquery&qnum=20) 388 [edit](JavaScript:doPopup('/search-web/cochrane/searchHistory?mode=editquery&qnum=20&searchKey=3be65063-a79e-4d5b-a2fb-3216df124862',%20400)) [delete](http://onlinelibrary.wiley.com/search-web/cochrane/searchHistory?mode=deletequery&qnum=20&uuid=3be65063-a79e-4d5b-a2fb-3216df124862&searchKey=3be65063-a79e-4d5b-a2fb-3216df124862)

#21 [Midwifery or midwives or midwife or Obstetrical Nursing](http://onlinelibrary.wiley.com/o/cochrane/searchHistory?mode=runquery&qnum=21) 1495 [edit](JavaScript:doPopup('/search-web/cochrane/searchHistory?mode=editquery&qnum=21&searchKey=3be65063-a79e-4d5b-a2fb-3216df124862',%20400)) [delete](http://onlinelibrary.wiley.com/search-web/cochrane/searchHistory?mode=deletequery&qnum=21&uuid=3be65063-a79e-4d5b-a2fb-3216df124862&searchKey=3be65063-a79e-4d5b-a2fb-3216df124862)

#22 [non-physician clinician* or non-physician clinician* or non physician clinician OR npc OR npcs](http://onlinelibrary.wiley.com/o/cochrane/searchHistory?mode=runquery&qnum=22) 2256 [edit](JavaScript:doPopup('/search-web/cochrane/searchHistory?mode=editquery&qnum=22&searchKey=3be65063-a79e-4d5b-a2fb-3216df124862',%20400)) [delete](http://onlinelibrary.wiley.com/search-web/cochrane/searchHistory?mode=deletequery&qnum=22&uuid=3be65063-a79e-4d5b-a2fb-3216df124862&searchKey=3be65063-a79e-4d5b-a2fb-3216df124862)

#23 [MeSH descriptor **Allied Health Personnel** explode all trees](http://onlinelibrary.wiley.com/o/cochrane/searchHistory?mode=runquery&qnum=23) 534 [edit](http://onlinelibrary.wiley.com/search-web/cochrane/searchHistory?mode=editquery&qnum=23&searchKey=3be65063-a79e-4d5b-a2fb-3216df124862) [delete](http://onlinelibrary.wiley.com/search-web/cochrane/searchHistory?mode=deletequery&qnum=23&uuid=3be65063-a79e-4d5b-a2fb-3216df124862&searchKey=3be65063-a79e-4d5b-a2fb-3216df124862)

#24 [MeSH descriptor **Peer Group** explode all trees](http://onlinelibrary.wiley.com/o/cochrane/searchHistory?mode=runquery&qnum=24) 653 [edit](http://onlinelibrary.wiley.com/search-web/cochrane/searchHistory?mode=editquery&qnum=24&searchKey=3be65063-a79e-4d5b-a2fb-3216df124862) [delete](http://onlinelibrary.wiley.com/search-web/cochrane/searchHistory?mode=deletequery&qnum=24&uuid=3be65063-a79e-4d5b-a2fb-3216df124862&searchKey=3be65063-a79e-4d5b-a2fb-3216df124862)

#25 [(lay or voluntary or volunteer* or untrained or unlicensed or nonprofessional* or non next professional*) NEAR/5 (worker* or visitor* or attendant* or aide or aides or support* or person* or helper* or carer* or caregiver* or care next giver* or consultant* or assistant* or staff or visit* or midwife or midwives):ti OR (lay or voluntary or volunteer* or untrained or unlicensed or nonprofessional* or non next professional*) NEAR/5 (worker* or visitor* or attendant* or aide or aides or support* or person* or helper* or carer* or caregiver* or care next giver* or consultant* or assistant* or staff or visit* or midwife or midwives):ab OR lay next volunteer*:ti OR lay next volunteer*:ab](http://onlinelibrary.wiley.com/o/cochrane/searchHistory?mode=runquery&qnum=25) 568 [edit](JavaScript:doPopup('/search-web/cochrane/searchHistory?mode=editquery&qnum=25&searchKey=3be65063-a79e-4d5b-a2fb-3216df124862',%20400)) [delete](http://onlinelibrary.wiley.com/search-web/cochrane/searchHistory?mode=deletequery&qnum=25&uuid=3be65063-a79e-4d5b-a2fb-3216df124862&searchKey=3be65063-a79e-4d5b-a2fb-3216df124862)

#26 [(paraprofessional* or paramedic or paramedics or paramedical NEXT worker* or paramedical NEXT personnel or allied NEXT health NEXT personnel or allied NEXT health NEXT worker* or support NEXT worker* or home NEXT health NEXT aide*):ti OR (paraprofessional* or paramedic or paramedics or paramedical NEXT worker* or paramedical NEXT personnel or allied NEXT health NEXT personnel or allied NEXT health NEXT worker* or support NEXT worker* or home next health next aide*):ab](http://onlinelibrary.wiley.com/o/cochrane/searchHistory?mode=runquery&qnum=26) 392 [edit](JavaScript:doPopup('/search-web/cochrane/searchHistory?mode=editquery&qnum=26&searchKey=3be65063-a79e-4d5b-a2fb-3216df124862',%20400)) [delete](http://onlinelibrary.wiley.com/search-web/cochrane/searchHistory?mode=deletequery&qnum=26&uuid=3be65063-a79e-4d5b-a2fb-3216df124862&searchKey=3be65063-a79e-4d5b-a2fb-3216df124862)

#27 [(trained NEAR/3 (volunteer* or (health next worker*) or mother*)):ti OR (trainedNEAR/3 (volunteer* or (health next worker*) or mother*)):ab](http://onlinelibrary.wiley.com/o/cochrane/searchHistory?mode=runquery&qnum=27) 42 [edit](JavaScript:doPopup('/search-web/cochrane/searchHistory?mode=editquery&qnum=27&searchKey=3be65063-a79e-4d5b-a2fb-3216df124862',%20400)) [delete](http://onlinelibrary.wiley.com/search-web/cochrane/searchHistory?mode=deletequery&qnum=27&uuid=3be65063-a79e-4d5b-a2fb-3216df124862&searchKey=3be65063-a79e-4d5b-a2fb-3216df124862)

#28 [(community or village*) NEAR/3 ((health next worker*) or (health next care next worker*) or (healthcare next worker*)):ti OR (community or village*) NEAR/3 ((health next worker*) or (health next care next worker*) or (healthcare next worker*)):ab](http://onlinelibrary.wiley.com/o/cochrane/searchHistory?mode=runquery&qnum=28) 168 [edit](JavaScript:doPopup('/search-web/cochrane/searchHistory?mode=editquery&qnum=28&searchKey=3be65063-a79e-4d5b-a2fb-3216df124862',%20400)) [delete](http://onlinelibrary.wiley.com/search-web/cochrane/searchHistory?mode=deletequery&qnum=28&uuid=3be65063-a79e-4d5b-a2fb-3216df124862&searchKey=3be65063-a79e-4d5b-a2fb-3216df124862)

#29 [(community NEAR/3 (volunteer* or aide or aides or support)):ti or (community NEAR/3 (volunteer* or aide or aides or support)):ab](http://onlinelibrary.wiley.com/o/cochrane/searchHistory?mode=runquery&qnum=29) 315 [edit](JavaScript:doPopup('/search-web/cochrane/searchHistory?mode=editquery&qnum=29&searchKey=3be65063-a79e-4d5b-a2fb-3216df124862',%20400)) [delete](http://onlinelibrary.wiley.com/search-web/cochrane/searchHistory?mode=deletequery&qnum=29&uuid=3be65063-a79e-4d5b-a2fb-3216df124862&searchKey=3be65063-a79e-4d5b-a2fb-3216df124862)

#30 [(birth or childbirth or labor or labour) NEXT (attendant* or assistant*):ti OR (birth or childbirth or labor or labour) NEXT (attendant* or assistant*):ab](http://onlinelibrary.wiley.com/o/cochrane/searchHistory?mode=runquery&qnum=30) 54 [edit](JavaScript:doPopup('/search-web/cochrane/searchHistory?mode=editquery&qnum=30&searchKey=3be65063-a79e-4d5b-a2fb-3216df124862',%20400)) [delete](http://onlinelibrary.wiley.com/search-web/cochrane/searchHistory?mode=deletequery&qnum=30&uuid=3be65063-a79e-4d5b-a2fb-3216df124862&searchKey=3be65063-a79e-4d5b-a2fb-3216df124862)

#31 [(doula* or douladural*):ti OR (doula* or douladural*):ab](http://onlinelibrary.wiley.com/o/cochrane/searchHistory?mode=runquery&qnum=31) 16 [edit](JavaScript:doPopup('/search-web/cochrane/searchHistory?mode=editquery&qnum=31&searchKey=3be65063-a79e-4d5b-a2fb-3216df124862',%20400)) [delete](http://onlinelibrary.wiley.com/search-web/cochrane/searchHistory?mode=deletequery&qnum=31&uuid=3be65063-a79e-4d5b-a2fb-3216df124862&searchKey=3be65063-a79e-4d5b-a2fb-3216df124862)

#32 [(monitrice*):ti OR (monitrice*):ab](http://onlinelibrary.wiley.com/o/cochrane/searchHistory?mode=runquery&qnum=32) 1 [edit](JavaScript:doPopup('/search-web/cochrane/searchHistory?mode=editquery&qnum=32&searchKey=3be65063-a79e-4d5b-a2fb-3216df124862',%20400)) [delete](http://onlinelibrary.wiley.com/search-web/cochrane/searchHistory?mode=deletequery&qnum=32&uuid=3be65063-a79e-4d5b-a2fb-3216df124862&searchKey=3be65063-a79e-4d5b-a2fb-3216df124862)

#33 [(peer NEXT (volunteer* or counsel* or support or intervention*)):ti OR (peer NEXT (volunteer* or counsel* or support or intervention*)):ab](http://onlinelibrary.wiley.com/o/cochrane/searchHistory?mode=runquery&qnum=33) 250 [edit](JavaScript:doPopup('/search-web/cochrane/searchHistory?mode=editquery&qnum=33&searchKey=3be65063-a79e-4d5b-a2fb-3216df124862',%20400)) [delete](http://onlinelibrary.wiley.com/search-web/cochrane/searchHistory?mode=deletequery&qnum=33&uuid=3be65063-a79e-4d5b-a2fb-3216df124862&searchKey=3be65063-a79e-4d5b-a2fb-3216df124862)

#34 [outreach:ti OR outreach:ab](http://onlinelibrary.wiley.com/o/cochrane/searchHistory?mode=runquery&qnum=34) 565 [edit](JavaScript:doPopup('/search-web/cochrane/searchHistory?mode=editquery&qnum=34&searchKey=3be65063-a79e-4d5b-a2fb-3216df124862',%20400)) [delete](http://onlinelibrary.wiley.com/search-web/cochrane/searchHistory?mode=deletequery&qnum=34&uuid=3be65063-a79e-4d5b-a2fb-3216df124862&searchKey=3be65063-a79e-4d5b-a2fb-3216df124862)

#35 [(church next based) NEAR/3 (intervention* or program* or counsel*):ti OR (church next based) NEAR/3 (intervention* or program* or counsel*):ab](http://onlinelibrary.wiley.com/o/cochrane/searchHistory?mode=runquery&qnum=35) 13 [edit](JavaScript:doPopup('/search-web/cochrane/searchHistory?mode=editquery&qnum=35&searchKey=3be65063-a79e-4d5b-a2fb-3216df124862',%20400)) [delete](http://onlinelibrary.wiley.com/search-web/cochrane/searchHistory?mode=deletequery&qnum=35&uuid=3be65063-a79e-4d5b-a2fb-3216df124862&searchKey=3be65063-a79e-4d5b-a2fb-3216df124862)

#36 [(linkworker* or (link next worker*)):ti OR (linkworker* or (link next worker*)):ab](http://onlinelibrary.wiley.com/o/cochrane/searchHistory?mode=runquery&qnum=36) 5 [edit](JavaScript:doPopup('/search-web/cochrane/searchHistory?mode=editquery&qnum=36&searchKey=3be65063-a79e-4d5b-a2fb-3216df124862',%20400)) [delete](http://onlinelibrary.wiley.com/search-web/cochrane/searchHistory?mode=deletequery&qnum=36&uuid=3be65063-a79e-4d5b-a2fb-3216df124862&searchKey=3be65063-a79e-4d5b-a2fb-3216df124862)

#37 [(barefoot next doctor*):ti OR (barefoot next doctor*):ab](http://onlinelibrary.wiley.com/o/cochrane/searchHistory?mode=runquery&qnum=37) 0 [edit](JavaScript:doPopup('/search-web/cochrane/searchHistory?mode=editquery&qnum=37&searchKey=3be65063-a79e-4d5b-a2fb-3216df124862',%20400)) [delete](http://onlinelibrary.wiley.com/search-web/cochrane/searchHistory?mode=deletequery&qnum=37&uuid=3be65063-a79e-4d5b-a2fb-3216df124862&searchKey=3be65063-a79e-4d5b-a2fb-3216df124862)

#38 [(home NEXT (care or aide or aides or nursing or support or intervention* or treatment* or visit*)):ti OR (home NEXT (care or aide or aides or nursing or support or intervention* or treatment* or visit*)):ab](http://onlinelibrary.wiley.com/o/cochrane/searchHistory?mode=runquery&qnum=38) 1886 [edit](JavaScript:doPopup('/search-web/cochrane/searchHistory?mode=editquery&qnum=38&searchKey=3be65063-a79e-4d5b-a2fb-3216df124862',%20400)) [delete](http://onlinelibrary.wiley.com/search-web/cochrane/searchHistory?mode=deletequery&qnum=38&uuid=3be65063-a79e-4d5b-a2fb-3216df124862&searchKey=3be65063-a79e-4d5b-a2fb-3216df124862)

#39 [(care OR aide OR aides OR nursing OR support OR intervention* OR treatment* OR visit*) NEAR/3 (lay or volunteer* or voluntary)](http://onlinelibrary.wiley.com/o/cochrane/searchHistory?mode=runquery&qnum=39) 1256 [edit](JavaScript:doPopup('/search-web/cochrane/searchHistory?mode=editquery&qnum=39&searchKey=3be65063-a79e-4d5b-a2fb-3216df124862',%20400)) [delete](http://onlinelibrary.wiley.com/search-web/cochrane/searchHistory?mode=deletequery&qnum=39&uuid=3be65063-a79e-4d5b-a2fb-3216df124862&searchKey=3be65063-a79e-4d5b-a2fb-3216df124862)

#40 [(#38 OR #39)](http://onlinelibrary.wiley.com/o/cochrane/searchHistory?mode=runquery&qnum=40) 3110 [edit](JavaScript:doPopup('/search-web/cochrane/searchHistory?mode=editquery&qnum=40&searchKey=3be65063-a79e-4d5b-a2fb-3216df124862',%20400)) [delete](http://onlinelibrary.wiley.com/search-web/cochrane/searchHistory?mode=deletequery&qnum=40&uuid=3be65063-a79e-4d5b-a2fb-3216df124862&searchKey=3be65063-a79e-4d5b-a2fb-3216df124862)

#41 [(#19 OR #20 OR #21 OR #23 OR #24 OR #25 OR #26 OR #27 OR #28 OR #29 OR #30 OR #31 OR #32 OR #33 OR #34 OR #35 OR #36 OR #37 OR #40)](http://onlinelibrary.wiley.com/o/cochrane/searchHistory?mode=runquery&qnum=41) 25845 [edit](JavaScript:doPopup('/search-web/cochrane/searchHistory?mode=editquery&qnum=41&searchKey=3be65063-a79e-4d5b-a2fb-3216df124862',%20400)) [delete](http://onlinelibrary.wiley.com/search-web/cochrane/searchHistory?mode=deletequery&qnum=41&uuid=3be65063-a79e-4d5b-a2fb-3216df124862&searchKey=3be65063-a79e-4d5b-a2fb-3216df124862)

#42 [(#41 AND #18)](http://onlinelibrary.wiley.com/o/cochrane/searchHistory?mode=runquery&qnum=42) 431 [edit](JavaScript:doPopup('/search-web/cochrane/searchHistory?mode=editquery&qnum=42&searchKey=3be65063-a79e-4d5b-a2fb-3216df124862',%20400)) [delete](http://onlinelibrary.wiley.com/search-web/cochrane/searchHistory?mode=deletequery&qnum=42&uuid=3be65063-a79e-4d5b-a2fb-3216df124862&searchKey=3be65063-a79e-4d5b-a2fb-3216df124862)

#43 [(Africa or Asia or Caribbean or "West Indies" or "South America" or "Latin America" or "Central America"):ti,ab,kw](http://onlinelibrary.wiley.com/o/cochrane/searchHistory?mode=runquery&qnum=43) 2961 [edit](JavaScript:doPopup('/search-web/cochrane/searchHistory?mode=editquery&qnum=43&searchKey=3be65063-a79e-4d5b-a2fb-3216df124862',%20400)) [delete](http://onlinelibrary.wiley.com/search-web/cochrane/searchHistory?mode=deletequery&qnum=43&uuid=3be65063-a79e-4d5b-a2fb-3216df124862&searchKey=3be65063-a79e-4d5b-a2fb-3216df124862)

#44 [(Afghanistan or Albania or Algeria or Angola or Antigua or Barbuda or Argentina or Armenia or Armenian or Aruba or Azerbaijan or Bahrain or Bangladesh or Barbados or Benin or Byelarus or Byelorussian or Belarus or Belorussian or Belorussia or Belize or Bhutan or Bolivia or Bosnia or Herzegovina or Hercegovina or Botswana or Brazil or Bulgaria or "Burkina Faso" or "Burkina Fasso" or "Upper Volta" or Burundi or Urundi or Cambodia or "Khmer Republic" or Kampuchea or Cameroon or Cameroons or Cameron or Camerons or "Cape Verde" or "Central African Republic" or Chad or Chile or China or Colombia or Comoros or "Comoro Islands" or Comores or Mayotte or Congo or Zaire or "Costa Rica" or "Cote d'Ivoire" or "Ivory Coast" or Croatia or Cuba or Cyprus or Czechoslovakia or "Czech Republic" or Slovakia or "Slovak Republic"):ti,ab,kw](http://onlinelibrary.wiley.com/o/cochrane/searchHistory?mode=runquery&qnum=44) 6059 [edit](JavaScript:doPopup('/search-web/cochrane/searchHistory?mode=editquery&qnum=44&searchKey=3be65063-a79e-4d5b-a2fb-3216df124862',%20400)) [delete](http://onlinelibrary.wiley.com/search-web/cochrane/searchHistory?mode=deletequery&qnum=44&uuid=3be65063-a79e-4d5b-a2fb-3216df124862&searchKey=3be65063-a79e-4d5b-a2fb-3216df124862)

#45 [(Djibouti or "French Somaliland" or Dominica or "Dominican Republic" or "East Timor" or "East Timur" or "Timor Leste" or Ecuador or Egypt or "United Arab Republic" or "El Salvador" or Eritrea or Estonia or Ethiopia or Fiji or Gabon or "Gabonese Republic" or Gambia or Gaza or Georgia or Georgian or Ghana or "Gold Coast" or Greece or Grenada or Guatemala or Guinea or Guam or Guiana or Guyana or Haiti or Honduras or Hungary or India or Maldives or Indonesia or Iran or Iraq or "Isle of Man" or Jamaica or Jordan or Kazakhstan or Kazakh or Kenya or Kiribati or Korea or Kosovo or Kyrgyzstan or Kirghizia or "Kyrgyz Republic" or Kirghiz or Kirgizstan or "Lao PDR" or Laos or Latvia or Lebanon or Lesotho or Basutoland or Liberia or Libya or Lithuania):ti,ab,kw](http://onlinelibrary.wiley.com/o/cochrane/searchHistory?mode=runquery&qnum=45) 8078 [edit](JavaScript:doPopup('/search-web/cochrane/searchHistory?mode=editquery&qnum=45&searchKey=3be65063-a79e-4d5b-a2fb-3216df124862',%20400)) [delete](http://onlinelibrary.wiley.com/search-web/cochrane/searchHistory?mode=deletequery&qnum=45&uuid=3be65063-a79e-4d5b-a2fb-3216df124862&searchKey=3be65063-a79e-4d5b-a2fb-3216df124862)

#46 [(Macedonia or Madagascar or "Malagasy Republic" or Malaysia or Malaya or Malay or Sabah or Sarawak or Malawi or Nyasaland or Mali or Malta or "Marshall Islands" or Mauritania or Mauritius or "Agalega Islands" or Mexico or Micronesia or "Middle East" or Moldova or Moldovia or Moldovian or Mongolia or Montenegro or Morocco or Ifni or Mozambique or Myanmar or Myanma or Burma or Namibia or Nepal or "Netherlands Antilles" or "New Caledonia" or Nicaragua or Niger or Nigeria or "Northern Mariana Islands" or Oman or Muscat or Pakistan or Palau or Palestine or Panama or Paraguay or Peru or Philippines or Philipines or Phillipines or Phillippines or Poland or Portugal or "Puerto Rico"):ti,ab,kw](http://onlinelibrary.wiley.com/o/cochrane/searchHistory?mode=runquery&qnum=46) 4167 [edit](JavaScript:doPopup('/search-web/cochrane/searchHistory?mode=editquery&qnum=46&searchKey=3be65063-a79e-4d5b-a2fb-3216df124862',%20400)) [delete](http://onlinelibrary.wiley.com/search-web/cochrane/searchHistory?mode=deletequery&qnum=46&uuid=3be65063-a79e-4d5b-a2fb-3216df124862&searchKey=3be65063-a79e-4d5b-a2fb-3216df124862)

#47 [(Romania or Rumania or Roumania or Russia or Russian or Rwanda or Ruanda or "Saint Kitts" or "St Kitts" or Nevis or "Saint Lucia" or "St Lucia" or "Saint Vincent" or "St Vincent" or Grenadines or Samoa or "Samoan Islands" or "Navigator Island" or "Navigator Islands" or "Sao Tome" or "Saudi Arabia" or Senegal or Serbia or Montenegro or Seychelles or "Sierra Leone" or Slovenia or "Sri Lanka" or Ceylon or "Solomon Islands" or Somalia or Sudan or Suriname or Surinam or Swaziland or Syria or Tajikistan or Tadzhikistan or Tadjikistan or Tadzhik or Tanzania or Thailand or Togo or "Togolese Republic" or Tonga or Trinidad or Tobago or Tunisia or Turkey or Turkmenistan or Turkmen or Uganda or Ukraine or Uruguay or USSR or "Soviet Union" or "Union of Soviet Socialist Republics" or Uzbekistan or Uzbek or Vanuatu or "New Hebrides" or Venezuela or Vietnam or "Viet Nam" or "West Bank" or Yemen or Yugoslavia or Zambia or Zimbabwe or Rhodesia):ti,ab,kw](http://onlinelibrary.wiley.com/o/cochrane/searchHistory?mode=runquery&qnum=47) 5029 [edit](JavaScript:doPopup('/search-web/cochrane/searchHistory?mode=editquery&qnum=47&searchKey=3be65063-a79e-4d5b-a2fb-3216df124862',%20400)) [delete](http://onlinelibrary.wiley.com/search-web/cochrane/searchHistory?mode=deletequery&qnum=47&uuid=3be65063-a79e-4d5b-a2fb-3216df124862&searchKey=3be65063-a79e-4d5b-a2fb-3216df124862)

#48 [(developing or less* NEXT developed or "under developed" or underdeveloped or "middle income" or low* NEXT income or underserved or "under served" or deprived or poor*) NEXT (countr* or nation* or population* or world):ti,ab,kw](http://onlinelibrary.wiley.com/o/cochrane/searchHistory?mode=runquery&qnum=48) 2366 [edit](JavaScript:doPopup('/search-web/cochrane/searchHistory?mode=editquery&qnum=48&searchKey=3be65063-a79e-4d5b-a2fb-3216df124862',%20400)) [delete](http://onlinelibrary.wiley.com/search-web/cochrane/searchHistory?mode=deletequery&qnum=48&uuid=3be65063-a79e-4d5b-a2fb-3216df124862&searchKey=3be65063-a79e-4d5b-a2fb-3216df124862)

#49 [(developing or less* NEXT developed or "under developed" or underdeveloped or "middle income" or low* NEXT income) NEXT (economy or economies):ti,ab,kw](http://onlinelibrary.wiley.com/o/cochrane/searchHistory?mode=runquery&qnum=49) 9 [edit](JavaScript:doPopup('/search-web/cochrane/searchHistory?mode=editquery&qnum=49&searchKey=3be65063-a79e-4d5b-a2fb-3216df124862',%20400)) [delete](http://onlinelibrary.wiley.com/search-web/cochrane/searchHistory?mode=deletequery&qnum=49&uuid=3be65063-a79e-4d5b-a2fb-3216df124862&searchKey=3be65063-a79e-4d5b-a2fb-3216df124862)

#50 [low* NEXT (gdp or gnp or "gross domestic" or "gross national"):ti,ab,kw](http://onlinelibrary.wiley.com/o/cochrane/searchHistory?mode=runquery&qnum=50) 21 [edit](JavaScript:doPopup('/search-web/cochrane/searchHistory?mode=editquery&qnum=50&searchKey=3be65063-a79e-4d5b-a2fb-3216df124862',%20400)) [delete](http://onlinelibrary.wiley.com/search-web/cochrane/searchHistory?mode=deletequery&qnum=50&uuid=3be65063-a79e-4d5b-a2fb-3216df124862&searchKey=3be65063-a79e-4d5b-a2fb-3216df124862)

#51 [(low NEAR/3 middle NEAR/3 countr*):ti,ab,kw](http://onlinelibrary.wiley.com/o/cochrane/searchHistory?mode=runquery&qnum=51) 6 [edit](JavaScript:doPopup('/search-web/cochrane/searchHistory?mode=editquery&qnum=51&searchKey=3be65063-a79e-4d5b-a2fb-3216df124862',%20400)) [delete](http://onlinelibrary.wiley.com/search-web/cochrane/searchHistory?mode=deletequery&qnum=51&uuid=3be65063-a79e-4d5b-a2fb-3216df124862&searchKey=3be65063-a79e-4d5b-a2fb-3216df124862)

#52 [(lmic or lmics or "third world" or "lami country" or "lami countries"):ti,ab,kw](http://onlinelibrary.wiley.com/o/cochrane/searchHistory?mode=runquery&qnum=52) 54 [edit](JavaScript:doPopup('/search-web/cochrane/searchHistory?mode=editquery&qnum=52&searchKey=3be65063-a79e-4d5b-a2fb-3216df124862',%20400)) [delete](http://onlinelibrary.wiley.com/search-web/cochrane/searchHistory?mode=deletequery&qnum=52&uuid=3be65063-a79e-4d5b-a2fb-3216df124862&searchKey=3be65063-a79e-4d5b-a2fb-3216df124862)

#53 [("transitional country" or "transitional countries"):ti,ab,kw](http://onlinelibrary.wiley.com/o/cochrane/searchHistory?mode=runquery&qnum=53) 0 [edit](JavaScript:doPopup('/search-web/cochrane/searchHistory?mode=editquery&qnum=53&searchKey=3be65063-a79e-4d5b-a2fb-3216df124862',%20400)) [delete](http://onlinelibrary.wiley.com/search-web/cochrane/searchHistory?mode=deletequery&qnum=53&uuid=3be65063-a79e-4d5b-a2fb-3216df124862&searchKey=3be65063-a79e-4d5b-a2fb-3216df124862)

#54 [(#43 OR #44 OR #45 OR #46 OR #47 OR #48 OR #49 OR #50 OR #51 OR #52 OR #53)](http://onlinelibrary.wiley.com/o/cochrane/searchHistory?mode=runquery&qnum=54) 24910 [edit](JavaScript:doPopup('/search-web/cochrane/searchHistory?mode=editquery&qnum=54&searchKey=3be65063-a79e-4d5b-a2fb-3216df124862',%20400)) [delete](http://onlinelibrary.wiley.com/search-web/cochrane/searchHistory?mode=deletequery&qnum=54&uuid=3be65063-a79e-4d5b-a2fb-3216df124862&searchKey=3be65063-a79e-4d5b-a2fb-3216df124862)

#55 [(#18 AND #41 AND #54)](http://onlinelibrary.wiley.com/o/cochrane/searchHistory?mode=runquery&qnum=55) 97 [edit](JavaScript:doPopup('/search-web/cochrane/searchHistory?mode=editquery&qnum=55&searchKey=3be65063-a79e-4d5b-a2fb-3216df124862',%20400)) [delete](http://onlinelibrary.wiley.com/search-web/cochrane/searchHistory?mode=deletequery&qnum=55&uuid=3be65063-a79e-4d5b-a2fb-3216df124862&searchKey=3be65063-a79e-4d5b-a2fb-3216df124862)

### 5. Cumulative Index of Nursing and Allied Health (CINAHL)

S1 TI ( (Lay OR lay OR lay volunteer OR community or extension OR lady ) N3 (worker OR health worker OR volunteer) OR lay health workers OR community workers OR community volunteers OR barefoot doctor OR doula OR doulas OR auxiliary worker? OR (health N3 assistants) OR auxiliary nurse? OR nurse auxiliary* OR nurse assistant* OR nurse midwife OR nurse midwives OR nurse* OR midwife OR midwives OR auxiliary nurse midwives OR traditional birth attendant? OR tba OR tbas OR non-specialist doctor? OR non clinician physician OR non-clinician physician? OR non-physician? OR mid-level provider? OR non physician? OR physician assistan* OR clinician? ) OR AB ( (Lay OR lay OR lay volunteer OR community or extension OR lady ) N3 (worker OR health worker OR volunteer) OR lay health workers OR community workers OR community volunteers OR barefoot doctor OR doula OR doulas OR auxiliary worker? OR (health N3 assistants) OR auxiliary nurse? OR nurse auxiliary* OR nurse assistant* OR nurse midwife OR nurse midwives OR nurse* OR midwife OR midwives OR auxiliary nurse midwives OR traditional birth attendant? OR tba OR tbas OR non-specialist doctor? OR non clinician physician OR non-clinician physician? OR non-physician? OR mid-level provider? OR non physician? OR physician assistan* OR clinician? )TI ( (Lay OR lay OR lay volunteer OR community or extension OR lady ) N3 (worker OR health worker OR volunteer) OR lay health workers OR community workers OR community volunteers OR barefoot doctor OR doula OR doulas OR auxiliary worker? OR (health N3 assistants) OR auxiliary nurse? OR nurse auxiliary* OR nurse assistant* OR nurse midwife OR nurse midwives OR nurse* OR midwife OR midwives OR auxiliary nurse midwives OR traditional birth attendant? OR tba OR tbas OR non-specialist doctor? (99768)

S2 (MH " Personnel, Unlicensed") OR (MH "Home Aides") OR (MH "Midwives+") OR (MH "Lay Midwives") OR (MH "Multiskilled Practitioners") OR (MH "Nurses+") OR (MH "Nurses by Educational Level+") OR (MH "Nurses by Role+") OR (MH "Advanced Practice Nurses+") OR (MH "Clinical Nurse Specialists") OR (MH "Nurse Anesthetists") OR (MH "Nurse Midwives") OR (MH "Nurse Practitioners+") OR (MH "Family Nurse Practitioners") OR (MH "Gerontologic Nurse Practitioners") OR (MH "OB-GYN Nurse Practitioners") OR (MH "Pediatric Nurse Practitioners+") OR (MH "Neonatal Nurse Practitioners") OR (MH "Associate Degree Nurses") OR (MH "Baccalaureate Nurses") OR (MH "Diploma Nurses") OR (MH "Doctorally Prepared Nurses") OR (MH "Masters-Prepared Nurses") OR (MH " Facility Administrators") OR (MH "Expert Clinicians+") OR (MH "Expert Nurses") OR (MH "Community Workers") OR (MH "Interns and Residents") OR (MH " Personnel, Minority+") OR (MH "Nurses, Minority") OR (MH " Manpower+") OR (MH "Nurse Administrators") OR (MH "Physician Executives") OR (MH "Administrative Personnel+") OR (MH "Clerical Personnel") OR (MH "Volunteer Workers") OR (MH "Rural Personnel") OR (MH "Nursing Staff, Hospital") OR (MH "Medical Staff, Hospital+") OR (MH "Personnel, Facility+") OR (MH "Allied Personnel+")(MH " Personnel, Unlicensed") OR (MH "Home Aides") OR (MH "Midwives+") OR (MH "Lay Midwives") OR (MH "Multiskilled Practitioners") OR (MH "Nurses+") OR (MH "Nurses by Educational Level+") OR (MH "Nurses by Role+") OR (MH "Advanced Practice Nurses+") OR (MH "Clinical Nurse Specialists") OR (MH "Nurse Anesthetists") OR (MH "Nurse Midwives") OR (MH "Nurse Practitioners+") OR (MH "Family Nurse Practitioners") OR (MH "Gerontologic Nurse Practitioners") OR (MH "OB-GYN Nurse Practitioners") OR (91160)

S3 S1 or S2 (157945)

S4 TI ( task shifting OR task sharing ) OR AB ( task shifting OR task sharing ) (83)

S5 S1 or S2 or S4

S6 (MH "Contraception+") OR "contraception" OR (MH "Contraceptive Agents, Male/SD") OR (MH "Contraceptive Agents+") OR (MH "Contraceptives, Oral+") OR (MH "Diaphragms, Contraceptive") OR (MH "Contraception Care (Saba CCC)") OR (MH "Contraceptive Devices+") OR (MH "Contraceptives, Oral Combined") OR (MH "Intrauterine Devices") OR (MH "Condoms") OR (MH "Levonorgestrel") OR (MH "Spermatocidal Agents") OR (MH "Sterilization, Sexual+") OR (MH "Sterilization, Tubal") OR (MH "Vasectomy") OR (MM "Surgery, Gynecologic") OR (MM "Surgery, Urologic, Male") OR (MH "Injections, Subcutaneous+") OR (MM "Injections") OR (MH "Injections, Intramuscular+") OR (MH "Drug Implants") OR "contraceptive implant"

S7 TI ( contraceptive agent? OR contraceptive? OR contraception OR contraceptive delivery OR condom? OR oral contraceptive* or intrauterine device? OR IUD? OR IUCD? OR intrauterine contraceptive device OR (implant? N3 contracept*) OR contraceptive implant? OR Norplant OR jadelle OR drug implant OR (implant* AND contracept*) OR (inject* AND contracept*) OR injectable contraceptive? OR Uniject OR (injection N5 contracept*) OR “sexual sterili?ation” OR tubal ligation OR “tubal sterilization” OR tubectomy OR “reproductive sterili?ation” OR vasectom* OR vas ligation OR vas occlusion OR male sterili?ation ) OR AB ( contraceptive agent? OR contraceptive? OR contraception OR contraceptive delivery OR condom? OR oral contraceptive* or intrauterine device? OR IUD? OR IUCD? OR intrauterine contraceptive device OR (implant? N3 contracept*) OR contraceptive implant? OR Norplant OR jadelle OR drug implant OR (implant* AND contracept*) OR (inject* AND contracept*) OR injectable contraceptive? OR Uniject OR (injection N5 contracept*) OR “sexual sterili?ation” OR tubal ligation OR “tubal sterilization” OR tubectomy OR “reproductive sterili?ation” OR vasectom* OR vas ligation OR vas occlusion OR “male sterili?ation” )

S8 S6 or S7

| S9 S5 and S8 |
| --- |
| S10 AB ( intervention* OR controlled OR (control W0 group*) OR compare OR compared OR (before N5 after) OR (pre N3 post) OR pretest OR "pre test" OR posttest OR "post test" OR quasiexperiment* OR (quasi W0 experiment*) OR evaluat* OR effect OR impact OR "time series" OR (time W0 point*) OR (repeated W0 measur*) ) OR TI ( intervention* OR controlled OR (control W0 group*) OR compare OR compared OR (before N5 after) OR (pre N3 post) OR pretest OR "pre test" OR posttest OR "post test" OR quasiexperiment* OR (quasi W0 experiment*) OR evaluat* OR effect OR impact OR "time series" OR (time W0 point*) or (repeated W0 measur*) )AB ( intervention* OR controlled OR (control W0 group*) OR compare OR compared OR (before N5 after) OR (pre N3 post) OR pretest OR "pre test" OR posttest OR "post test" OR quasiexperiment* OR (quasi W0 experiment*) OR evaluat* OR effect OR impact OR "time series" OR (time W0 point*) OR (repeated W0 measur*) ) OR TI ( intervention* OR controlled OR (control W0 group*) OR compare OR compared OR (before N5 after) OR (pre N3 post) OR pretest OR "pre test" OR posttest OR "post test" OR quasiexperiment |
| S11 TI trial or multicentre or multicenter or "multi centre" or "multi center" |
| S12 TI ( randomis* OR randomiz* OR randomly OR (random W0 allocat*) ) OR AB ( randomis* OR randomiz* OR randomly OR (random W0 allocat*) ) |
| S13 MH Services Research |
| S14 MH Multicenter Studies |
| S15 MH Quasi-Experimental Studies+ |
| S16 MH Pretest-Posttest Design+ |
| S17 MH Experimental Studies |
| S18 MH Nonrandomized Trials |
| S19 MH Intervention Trials |
| S20 MH Clinical Trials |
| S21 MH Randomized Controlled Trials |
| S22 PT research |
| S23 PT clinical trial |

S24 S10 OR S11 OR S12 OR S13 OR S14 OR S15 OR S16 OR S17 OR S18 OR S19 OR S20 OR S21 OR S22 OR S23

S25 S9 and S24 (174)

### 6. Global Health Library (WHOLIS and Regional Databases)

("Contraceptive Agents" OR "Hormones, Hormone Substitutes, and Hormone Antagonists" OR "Surgical Procedures, Minor") AND ("Allied Health Occupations" OR "Nursing" OR "Nursing, Practical" OR "Health Manpower" OR "Health Personnel" OR "Health Occupations" OR "Voluntary Workers" OR "Women" OR "Health Manpower")
