# Supplementary material for: Optimizing the delivery of contraceptives in low- and middle-income countries through task shifting: a systematic review of effectiveness and safety
Source: Reprod Health. 2015 Apr 1;12:27. doi: 10.1186/s12978-015-0002-2 (PMC4392779; doi:10.1186/s12978-015-0002-2)
Supplement: Additional file 3: — Cochrane data extraction form. [file 12978_2015_2_MOESM3_ESM.docx]

# Additional file 3: Cochrane data extraction form

1. Objectives of the study
2. Participant information, including on health worker cadres in the intervention and comparison groups, and on recipients
3. Training information of health workers
4. Demographic details of recipients
5. Number of settings
6. Healthcare setting (home, primary care facility, hospital setting or other), geographic setting (rural, urban) and country
7. Study design (RCT, NRCT, CBA and ITS)
8. Contraceptive delivery performed
9. Outcomes assessed and timing of the outcome assessment (follow-up)
