# Supplementary material for: Optimizing the delivery of contraceptives in low- and middle-income countries through task shifting: a systematic review of effectiveness and safety
Source: Reprod Health. 2015 Apr 1;12:27. doi: 10.1186/s12978-015-0002-2 (PMC4392779; doi:10.1186/s12978-015-0002-2)
Supplement: Additional file 4: — Characteristics of the included studies. [file 12978_2015_2_MOESM4_ESM.docx]

## Additional file 4: Characteristics of the included studies

**Lassner *et al*. 1995**

| **Study design** | Randomized controlled trial | | |  |
| --- | --- | --- | --- | --- |
| **Participants** | HEALTH WORKER: Nurses with university degree or without (‘technical nurses’), COMPARISON: Doctors  TRAINING: For nurses with university degree, the training lasted for 6 weeks, including 2 weeks of theory and 4 weeks of practice. Training for technical nurses (and occasionally auxiliary nurses) usually lasted 8 weeks, including 2 weeks of theory and 6 weeks of practice. A minimum of 10 solo insertions were required during training.  PARTICIPANTS: All women who were eligible to receive an IUD and who voluntarily requested IUD insertion at the Center for Research on Integrated Maternal and Child Care central clinic, Brazil. Mean parity was 1.59 and significantly dissimilar in both groups (doctors: 1.66 vs. nurses: 1.52). Mean age was similar (27.6 years). Education was dissimilar in the two groups: 6.1 years (doctors) vs. 6.5 years (nurses). Also women reporting previous PID or STDs were significantly dissimilar: 11.2% (doctors) vs. 4.1% (nurses).  TOTAL: 1711, INTERVENTION: 851, CONTROL: 860 | | |  |
| **Intervention** | IUD insertion with 1,6 and 12 month follow-up from November 1984 to April 1986 | | |  |
| **Setting** | HEALTHCARE SETTING: Hospital setting (the main clinic of the Center of Research on Integrated Maternal and Child Care)  GEOGRAPHICAL SETTING: Rio de Janeiro, Brazil | | |  |
| **Outcomes** | SAFETY AND EFFECTIVENESS:  **Insertion failure rate**   - Nulliparous women: (RR 3.41, 95% CI 1.18 to 9.85, p=0.0237) - Multiparous women: (RR 1.66, 95% CI 0.65 to 4.25, p=0.2939)   **Complication rate during insertion**: 1.8% and 1.7% for nurses and doctors, respectively (RR 1.01, 95% CI 0.50 to 2.05, p=0.9768),  **Expulsion rate**: RR 0.93, 95% CI 0.57 to 1.52, p=0.7786  **Unintended pregnancy rate**: RR 0.75, 95%CI 0.26 to 2.14, p=0.5856  **Pain during insertion**: RR=0.65, 95%CI 0.48 to 0.89, p=0.0069  **Removal rate**: RR 0.71, 95% CI 0.47 to 1.06, p=0.0955  COVERAGE:  **Continuation rate (at 12 months)**: RR 0.99, 95% CI 0.93 to 1.06, p=0.7512 | | |  |
| **Risk of bias** | | | | |
| **Item** | | **Authors’ judgement** | **Support for judgement** | |
| Random sequence generation (selection bias) | | UNCLEAR | Women were randomly assigned to have an IUD inserted by one of the 11 doctors or 13 nurses at the clinic. The method of randomization used was not specified. | |
| Allocation concealment (selection bias) | | UNCLEAR | Concealment not clearly specified. | |
| Baseline characteristics similar (recruitment bias) | | HIGH RISK | Quote: “Some socio-demographic characteristics and medical conditions of the two groups were significantly dissimilar” despite randomization. | |
| Incomplete outcome data (attrition bias)  = Follow-up of patients or episodes of care | | HIGH RISK | Quote: “Partly because not all cases were actively followed up, 121 of the 1672 women with successfully inserted IUDs never returned to the clinic. Those who never returned (representing 7.2% of the overall sample, 8.1% of the doctors’ clients and 6.3% of the nurses’ clients) were excluded from the analysis. In the life-table analysis, 46% of all the cases (47.2% of the doctors’ clients and 44.7% of the nurses’ clients) were censored at less than one year because they were lost to follow-up However, 62.0% of them were observed for more than 6 months. So life‑table analysis excluded 7.2% of women who never returned, but included the 46% who were lost to follow-up, the results of that analysis may not reflect the true levels of IUD use‑effectiveness”. However, not valid for immediate results collected at IUD insertion. | |
| Blinded assessment of primary outcomes (protection against detection) bias | | LOW RISK | Objective outcomes: failure rate, pregnancy, expulsion rate, complication at insertion | |
|  |  | HIGH RISK | Side-effects, pain at insertion | |
| Adequate protection against contamination | | UNCLEAR | The control and intervention groups received care in the same clinic and it may therefore be possible that clients in the control group also received the intervention. Whether this did occur, however, is unclear. | |
| Selective reporting (reporting bias) | | UNCLEAR | No life tables and data shown for 1 and 6 months follow-up, although follow-ups were reported to be held at 1,6, and 12 months. Data at 12 month follow-up were provided in the study. | |
| Other bias | | LOW | The study seems to be free of other sources of bias. | |

**Einhorn *et al*. 1977**

| **Study design** | Randomized controlled trial |
| --- | --- |
| **Participants** | HEALTH WORKER: Licensed nurses (comparable to registered nurses in, for example, the USA) with standard nursing training, COMPARISON: Doctors  TRAINING: Licensed nurses received special training provided by PROFAMILIA (International Planned Parenthood Federation affiliate) in the delivery of general gynaecological and FP services to women. In the PROFAMILIA clinics, licensed nurses prescribe oral contraceptives, insert IUDs, perform breast, pelvic, speculum, and abdominal examinations, and treat vaginitis.  PARTICIPANTS: All women who came for a first visit during 6 weeks following 1 May 1975. The mean age was 25.7 years, the mean number of living children 2.3. 62.4% of the women had at least elementary school education.  TOTAL: 591, INTERVENTION: 279, CONTROL: 321 |
| **Interventions** | IUD insertion with 1, 6 and 12 month follow-up, after recruitment during a six‑week period, from 1 May 1975 to 15 June 1975. Data are, however, only available for 9 months of follow-up. |
| **Setting** | HEALTHCARE SETTING: Hospital setting (the main clinic of PROFAMILIA – the International Planned Parenthood Federation affiliate)  GEOGRAPHICAL SETTING: Bogotà, Colombia |
| **Outcomes** | SAFETY AND EFFECTIVENESS:  **Complication rate** during insertion (perforation of the uterus): No complications reported for either of the groups.  **Unintended pregnancy rate**: RR 0.40, 95% CI 0.04 to 3.79, p=0.4216  **Removal rate:** RR 1.75, 95% CI 0.93 to 3.40, p=0.0933  COVERAGE:  **Continuation rate (at 9 months)**: RR 0.98, 95%CI 0.91 to 1.05, p=0.5189 |

| **Risk of bias** | | |
| --- | --- | --- |
| **Item** | **Authors’ judgement** | **Support for judgement** |
| Random sequence generation | HIGH RISK | All new clients were randomly assigned to either a family planning nurse or doctor for the initial physical examination through an even/odd systematic numbering procedure. |
| Allocation concealment | HIGH RISK | Bias in the nurses’ group towards women who had never had a pregnancy. Quote: “there is a possibility that for cultural reasons these women were assigned purposefully to nurses by the interviewers”. |
| Baseline characteristics similar | UNCLEAR | Data is not available separately for the IUD clients. |
| Incomplete outcome data (attrition bias) | HIGH RISK | Loss to follow-up in both groups was similar, but very high. |
| Blinded assessment of primary outcomes (protection against detection bias) | LOW RISK | All outcomes objective |
| Adequate protection against contamination | UNCLEAR | Control and intervention groups received care in the same clinic and there is a possibility that clients in the control group might have received the intervention, but this is not clear. |
| Selective reporting (reporting bias) | HIGH RISK | No data on 12 month follow-up available. |
| Other bias | LOW | The study seems to be free of other sources of bias. |

**Eren *et al.* 1983, Study A**

| **Study design** | Randomized controlled trial |
| --- | --- |
| **Participants** | HEALTH WORKER: 14 auxiliary nurse-midwives, COMPARISON: 8 doctors  TRAINING: Trained to provide IUD services using the WHO manual and specially‑designed WHO checklists for the diagnosis and management of contraindications and complications of IUD use. Practical instruction was given in history taking, client counselling, pelvic examination, IUD insertion, and the continuing care of IUD users. When both the instructors and the trainees were confident that a satisfactory standard of competence had been achieved, the nurse-midwives were allowed to provide IUD services under normal programme circumstances. (Eren 1983 p 44, paragraph 1)  PARTICIPANTS: All interested women in the assessed villages.  TOTAL: 548, INTERVENTION: 278, CONTROL: 270 |
| **Interventions** | IUD insertion by auxiliary nurse-midwives compared to doctors, with 1, 3, 6, 9 and 12 month follow-up in a period of 3 years (1976-1979). |
| **Setting** | HEALTH CARE SETTING: Primary care setting (community level)  GEOGRAPHICAL SETTING: The Cubuk District, north of Ankara, Turkey |
| **Outcomes** | SAFETY AND EFFECTIVENESS:  **Expulsion rate**: RR 1.22, 95 % CI 0.65 to 2.27  **Unintended pregnancy rate:** RR 0.93, 95% CI 0.30 to 2.83  **Referral rate during IUD insertion:** RR 0.64, 95% CI 0.38 to 1.08, p=0.0518 (Providers of care referred, either because of preconditions of the patient identified during examination , i.e. suspected pregnancy, suspected pelvic inflammatory disease, cervicitis and erosion and conditions interfering with IUD insertion, e.g. prolapsed uterus, cervical incompetence)  **Referral rate after IUD insertion:** RR 1.23, 95% CI 0.65 to 2.36, p=0.5222 (the most common conditions referred were pregnancy, bleeding problems, suspected pelvic inflammatory disease, and missing IUD tail)  COVERAGE:  **Continuation rate** (at 9 months, we calculated from total discontinuations): RR 0.98, 95% CI 0.85 to 1.12, p= 0.7227 |

**Eren *et al.* 1983, Study B**

| **Study design** | Randomized controlled trial |
| --- | --- |
| **Participants** | HEALTH WORKER: 4 auxiliary nurse-midwives, COMPARISON: 2 doctors  TRAINING: Trained to provide IUD services using the WHO manual and specially‑designed WHO checklists for the diagnosis and management of contraindications and complications of IUD use. Practical instruction was given in history taking, client counselling, pelvic examination, IUD insertion, and the continuing care of IUD users. When both the instructors and the trainees were confident that a satisfactory standard of competence had been achieved, the nurse-midwives were allowed to provide IUD services under normal programme circumstances. (Eren 1983, p 44 paragraph 1).  PARTICIPANTS: Women 1-3 days postpartum  TOTAL: 510, INTERVENTION: 258, CONTROL: 252 |
| **Interventions** | IUD insertion with 1, 3, 6, 9 and 12 month follow-up in between 3 years (1976‑1979) |
| **Setting** | HEALTHCARE SETTING: Hospital setting (The Jose Fabella Memorial Hospital)  GEOGRAPHICAL SETTING: Manila, Philippines |
| **Outcomes** | SAFETY AND EFFECTIVENESS:  **Expulsion rate**: RR 0.64, 95% CI 0.38 to 1.10, p=0.1046  **Unintended pregnancy rate**: RR 1.00, 95%CI 0.25 to 3.94, p=0.9955  **Removal rate**: RR 1.62, 95% CI 0.68 to 3.84, p=0.2244  **Referral rate during IUD insertion**: RR 3.42, 95% CI 0.72 to 16.30, p=0.0967 (very small number of referrals in both groups for auxiliary nurses and doctors respectively (7/258 and 2/252)). Providers of care referred, either because of preconditions of the patient they diagnosed during examination (postpartum conditions such as an infected episiotomy or anaemia) or because they failed to insert the IUD.  **Referral rate after IUD insertion**: RR 2.16, 95% CI 0.83 to 5.59, p=0.1130  COVERAGE:  **Continuation rate (at 12 months**, we calculated from total discontinuations):  RR 01.09, 95% CI 1.00 to 1.19, p=0.0565 |

| **Risk of bias** | | |
| --- | --- | --- |
| **Item** | **Authors’ judgement** | **Support for judgement** |
| Random sequence generation | UNCLEAR | Volunteers were randomly allocated to auxiliary nurse-midwives or doctors (Eren 1983, page 44)  No information about *how* random selection was carried out. |
| Allocation concealment (selection bias) | UNCLEAR | Volunteers were randomly allocated to auxiliary nurse-midwives or doctors (Eren 1983, page 44) |
| Baseline characteristics similar (recruitment bias) | LOW RISK | The randomization procedures resulted in comparable distribution of age and parity among women in the doctor and the auxiliary nurse-midwife groups (Eren 1983, page 45, Table 1). |
| Incomplete outcome data (attrition bias) | LOW RISK | Loss to follow-up in setting 1: 9.7% for the intervention group and 9.2% for the control group. In setting 2: 2% for the intervention group and 6.4% for the control group (Eren 1983, page 45, Table 2). |
| Blinding of participants, personnel and outcome assessors (performance and detection bias): primary outcomes | LOW RISK | The following outcomes were objective: expulsion rate; pregnancy rate; continuation rate |
|  | UNCLEAR | The objectivity of the following outcomes is unclear: initial referral rate; referral during follow-up |
| Adequate protection against contamination | UNCLEAR | The intervention group and the control group were recruited from the same community |
| Selective reporting (reporting bias) | UNCLEAR | Doctors and nurse-midwives followed-up their own patients, which might have led to under-reporting of complications (only one follow-up examination was carried out by an independent doctor who did not know in which group the patient was). (Eren 1983, page 44, paragraph 7) |
| Other bias | LOW | The study seems to be free of other sources of bias. |

**Dusitsin *et al.* 1980**

| **Study design** | Randomized controlled trial |
| --- | --- |
| **Participants** | HEALTH WORKER: 5 nurse-midwives with more than 1-year of operation room training. The nurse-midwives in the study had received full nursing training, and an additional 18 months of midwifery training. They were therefore regarded as ‘midwives’ for the purpose of this review and referred to as such. COMPARISON: 3 doctors.  TRAINING: 12 weeks – trainees were taught pelvic and abdominal anatomy, and the principles of anaesthesia and postpartum tubal ligation. Animal tissue was used for practical training. After the preliminary phase, the trainees assisted gynaecologists with 3-5 cases. If a gynaecologist regarded a nurse-midwife as competent, the person was allowed to perform 20 operations under the supervision of a doctor. When trainees were judged to be competent, they were allowed to participate in the research study.  PARTICIPANTS: Informed volunteer subjects who requested sterilization before delivery. Healthy women with a normal vaginal delivery, no postpartum complications and no history of previous abdominal surgery. Characteristics: mean age: 28.0 (NM) and 18.4, mean body weight: 47.2 (SE 0.518) and 26.7 (0.434), proportion with episiotomy 20.3% vs. 24.8%. Statistically significant difference for mean thickness of abdominal fat (in cm): 2.2 (SE 0.075)vs. 1.9 (0.058).  TOTAL: 292, INTERVENTION: 143, CONTROL: 149 |
| **Interventions** | Performance of postpartum tubal ligation with follow-up at 5 days and 6 weeks after the operation (assessment of independent observer) |
| **Setting** | HEALTHCARE SETTING: Hospital setting (health centre)  GEOGRAPHICAL SETTING: Khon Kaen Province, Thailand |
| **Outcomes** | SAFETY AND EFFECTIVENESS:  **Complications rates during surgery** (due to thick abdominal fat, tubal adhesion, dextroverted uterus and inadequate sedation/analgesia): RR = 2.12, 95% CI 0.64 to 6.88, p=0.1895  **Postoperative complications** (mild pyrexia, respiratory infection, cystitis, wound breakdown): RR=1.16, 95% CI 0.48 to 2.66, p=0.7417  **Duration of operation**: Mean time = 18.5 minutes (SE 0.41) (nurses) vs. 11.9 minutes SE 0.32) (doctors), mean difference = 6.60 minutes, 95%, CI 5.58 to 7.62, p<0.0001 |

| **Risk of bias** | | |
| --- | --- | --- |
| **Item** | **Authors’ judgement** | **Support for judgement** |
| Random sequence generation | UNCLEAR | Method of sequence generation not specified. |
| Allocation concealment | UNCLEAR | Concealment of allocation not specified. |
| Baseline characteristics similar | HIGH | Quote: “The nurse-midwives’ patients were slightly heavier and had a significantly thicker abdominal layer of fat than did the doctor’s patients”. |
| Incomplete outcome data (attrition bias) | UNCLEAR | No information about loss to follow-up. |
| Blinding | LOW | Objective outcomes and independent assessment of outcomes.  Quote: “5 days and 6 weeks postoperatively an independent observer, who did not know whether a nurse or a doctor had performed the procedure, assessed the patients.” |
| Adequate protection against contamination | UNCLEAR | Not specified, but at same health centre, so bias cannot be excluded. |
| Selective reporting (reporting bias) | UNCLEAR | No data reported or tables shown for outcomes. |
| Other bias | UNCLEAR | It is unclear if there were any other sources of bias due to limited reporting of study design and results. |

**Bunyaratavej *et al*. 1981**

| **Study design** | Randomized controlled trial |
| --- | --- |
| **Participants** | HEALTH WORKER: Medical students in their third year of training without clinical experience, COMPARISON: Surgeons (members of the teaching staff at the Department of Surgery with a surgery residency training of not less than 3 years following medical school and internship.)  TRAINING: The trainees were taught male reproductive anatomy and physiology, principles of local anaesthesia, and vasectomy. Animal tissues were used for practical experience in the handling of instruments, tying ligatures, and dissection. After this preliminary phase, the trainees assisted surgeons with 5-10 vasectomies. If the surgeons considered the students to be competent, the students were then allowed to perform a minimum of 20 operations under the supervision of a surgeon.  PARTICIPANTS: Informed volunteer subjects who had requested sterilization. The 2 groups were comparable in terms of their socio-demographic characteristics, such as age and parity (No details listed).  TOTAL: 463, INTERVENTION: 276, CONTROL: 187 |
| **Interventions** | 10 students were selected at random to perform vasectomies (using the two-incisions technique). All medical students performed the operation with the assistance of another student, and a surgeon was always available in case of emergency. |
| **Setting** | HEALTHCARE SETTING: Hospital setting (Chulalongkorn Hospital)  GEOGRAPHICAL SETTING: Bangkok, Thailand |
| **Outcomes** | SAFETY AND EFFECTIVENESS:  **Complication rates during surgery** (errors in identifying and resecting the vas deferens): None observed among both groups  **Early post-operative complication rates** (within 7 days, identified as bleeding – requiring no treatment, requiring evacuation of blood clot; infection mild/superficial requiring no antibiotics, moderately severe requiring antibiotic treatment): RR 0.78, 95% CI 0.31 to 1.99, p=0.5536  **Postoperative oligospermia rates** (after 3 months, defined as more than 10,000/ml): RR = 2.59, 95% CI 0.87 to 7.70, p=0.0865 |

| **Risk of bias** | | | |
| --- | --- | --- | --- |
| **Item** | **Authors’ judgement** | **Support for judgement** | |
| Random sequence generation (selection bias) | UNCLEAR | Randomization of cases was done on a daily basis in the clinic, three students and two doctors were present. | |
| Allocation concealment (selection bias) | UNCLEAR | No information | |
| Baseline characteristics similar (recruitment bias) | LOW | The two groups had similar socio-demographic characteristics such as age and number of children. | |
| Incomplete outcome data (attrition bias) | LOW | Within 7 days:  Students: 7/276 = 2.5% | Surgeons: 0 /187 = 0% |
|  | HIGH | After 3 months:  83/276 = 30% | 46/187 = 24.5% |
| Blinding | LOW | Objective outcomes: Bleeding, infection, oligospermia assessed by an independent observer who did not know if a student or surgeon had performed the procedure | |
| Adequate protection against contamination | UNCLEAR | In the same hospital, a surgeon was available during the operation in case of an emergency. It is therefore possible that some contamination occurred, but whether this occurred is unclear. | |
| Selective reporting | LOW | There is a low possibility of selective reporting. | |
| Other bias | LOW | The study seems to be free of other sources of bias. | |
