# Supplementary material for: Optimizing the delivery of contraceptives in low- and middle-income countries through task shifting: a systematic review of effectiveness and safety
Source: Reprod Health. 2015 Apr 1;12:27. doi: 10.1186/s12978-015-0002-2 (PMC4392779; doi:10.1186/s12978-015-0002-2)
Supplement: Additional file 5: — Characteristics of the excluded studies. [file 12978_2015_2_MOESM5_ESM.docx]

# Additional file 5: Characteristics of the excluded studies

| **Study** | **Reason for Exclusion** |
| --- | --- |
| Affandi B, Prihartono J, Lubis F, Sutedi H and Samil RS [1] | Study design: Comparative cohort study. Only one site and the measurement (insertion time, complications etc.) took place only after intervention, excluding the possibility of a CBA. A NRCT study design was excluded because no allocation to control or study group was performed. |
| Arbab AA, McNamara R, Lauro D and Aziz FA [2] | Study design: Retrospective cohort study without control group |
| Bahamondes L, Marchi NM, Nakagava HM, de Melo ML, Cristofoletti Mde L, Pellini E, Scozzafave RH and Petta C [3] | Study design: Observational study of women self-injecting contraceptive |
| Bang S, Song SW and Choi CH [4] | Study design: Prospective comparative cohort study. Two sites with one nurse each delivering the IUD, but there is only one measurement of the outcomes of contraceptive delivery. No allocation by the investigators to the different interventions was made. Thus, neither a NRCT, CBA or ITS according to EPOC standards is possible. |
| Bayan F, Cruz AF and Sabino RV [5] | Full text and abstract not found |
| Chilopora G, Pereira C, Kamwendo F, Chimbiri A, Malunga E and Bergstrom S [6] | Study design: Prospective cohort study |
| Chowdhury S and Chowdhury Z [7] | Study design: Lack of information on methods, retrospective comparative cohort study |
| Diaz M, Faundes A, Marchi NM, Espejo X, Diaz J [8] | Study design: Retrospective cohort study |
| Fisher AA and de Silva V [9] | Study used teams (midwives and satisfied IUD acceptors). The objective of the study was to increase acceptance and use [of IUDs?] through promotional activities for which it was not possible to distinguish the safety and effectiveness of task-shifting for IUD insertion specifically. |
| Fongsri A and McDaniel EB [10] | Full text not found |
| Ghorbani FS [11] | Study design: No comparison group, lack of information about methods |
| Hardy E, Herud K [12] | Intervention: Did not assess task shifting between cadres |
| Hoke TH, Wheeler SB, Lynd K, Green MS, Razafindravony BH, Rasamihajamanana E and Blumenthal PD [13] | Health worker cadre: LHWs providing injections |
| Kanchanasinith K, Piyapinyo P, Pitaktepsombati P, Vibulsresth S, Gates DS, Janowitz B and Robbins M [14] | Study design: Prospective comparative cohort study. During the study period 820 women who came to the 15 different hospitals were either operated by a nurse-midwife or a physician, without purposeful allocation to the different interventions. The outcomes were then compared. Thus, this excludes any EPOC study design, such as a CBA, NRCT or ITS |
| Kim KS, Arshat H, Jali AHJ, Sucm E and Suhcdmi A [15] | Study design: Retrospective study based on case records |
| Koetsawang S VS, Satayapan S, Srisupanditi S, Apimas S [16] | Study design: Prospective comparative cohort study. The outcomes of postpartum sterilization performed by nurses were compared to the outcomes of a group of physicians that had performed sterilization. This design excludes an RCT, CBA or ITS study. As no allocation of the investigator to an intervention took place, an NRCT can be excluded as well. |
| Loghmani M and Mitra M [17] | Study design: Retrospective comparative cohort study |
| \| Marangoni P, Pozo A, Faundes A [18] \| \| --- \| | Study design: Retrospective comparative cohort study |
| Pastene L, Rivera M, Zipper J, Medel M and Thomas M [19] | Study design: Prospective comparative cohort study. Subjects were not randomly assigned to either group. No allocation to either intervention or control group took place; when a physician was in attendance at the hospital he performed the IUD insertions, otherwise nurse-midwives performed them. Thus, this study design is neither an NRCT nor any other study design as defined by the EPOC group. |
| Prata N, Gessessew A, Cartwright A and Fraser A [20] | Population and intervention: Lay health workers give injectable contraceptives. Additionally, the study compared one kind of lay health worker with another and therefore did not assess task shifting between cadres. |
| Ramos R and Apelo RA [21] | Study design: Prospective comparative cohort study. No allocation to any group was described, excluding NRCT as a possible study design. |
| Ronaghy HA, Zeighami B, Zeighami E and Nayeri F [22] | Study design: “Retrospective comparison”🡪 records/data were compared |
| Satyapan S, Varakamin S, Suwannus P, Chalapati S, Onthuam Y and Dusitsin N [23] | Study design: No comparison group, prospective cohort study |
| Siswosudarmo R [24] | Study design: Comparative cohort study – observational prospective study in which implant insertions of midwives and doctors were observed and afterwards compared. No allocation to the study or control group was observed. |
| Vaillant HW, Cummins GT, Richart RM and Barron BA [25] | Study design: Prospective comparative cohort study. Insertion by a doctor or nurse-midwife depended on the personnel available. Thus, no allocation to either control or study group was made, excluding the possibility of an NRCT study design and any other study of EPOC standards. |
| Villanueva Y, Mendoza I, Aguilar C, Rodríguez S and Vernon R [26] | Study design: “Single-group post-evaluation” – observational retrospective cohort study without comparison |
| Wright NH, Sujpluem C, Rosenfield AG, Varakamin S [27] | Study design: Retrospective comparative cohort study |
| Zhou SW and Chi IC [28] | Study design: Data set analysis – observational study |

**References**

1. Affandi B, Prihartono J, Lubis F, Sutedi H, Samil RS: **Insertion and removal of Norplant contraceptive implants by physicians and nonphysicians in an Indonesian clinic.** *Studies in family planning* 1987, **18:**302-306.

2. Arbab AA, McNamara R, Lauro D, Aziz FA: **Expanded services for intrauterine contraception in Sudan.** *East African medical journal* 1991, **68:**70-73.

3. Bahamondes L, Marchi NM, Nakagava HM, de Melo ML, Cristofoletti Mde L, Pellini E, Scozzafave RH, Petta C: **Self-administration with UniJect of the once-a-month injectable contraceptive Cyclofem.** *Contraception* 1997, **56:**301-304.

4. Bang S, Song SW, Choi CH: **Improving Access to the IUD: Experiments in Koyang, Korea.** *Studies in family planning* 1968, **1:**4-11.

5. Bayan F, Cruz AF, Sabino RV: **An evaluative study of nurses and midwives trained in comprehensive family planning service in the Philippines.** 1981.

6. Chilopora G, Pereira C, Kamwendo F, Chimbiri A, Malunga E, Bergstrom S: **Postoperative outcome of caesarean sections and other major emergency obstetric surgery by clinical officers and medical officers in Malawi.** *Human resources for health* 2007, **5:**17.

7. Chowdhury S, Chowdhury Z: **Tubectomy by paraprofessional surgeons in rural Bangladesh.** *Lancet* 1975, **2:**567-569.

8. Díaz M, Faúndes A, Marchi NM, Díaz J: **Comparación Del desempeno Del DIU TCU 200B insertado por médicos o enfermeras**. *Revista Iberoamericana de Fertilidad* 1992, 9(5):53-8.

9. Fisher AA, de Silva V: **Satisfied IUD acceptors as family planning motivators in Sri Lanka.** *Studies in family planning* 1986, **17:**235-242.

10. Fongsri A, McDaniel EB: **Use of nurse-midwives for minilap sterilization.** *Concern* 1979, **15:**13-15.

11. Ghorbani FS: **The use of paramedics in family planning services in Iran.** *International journal of gynaecology and obstetrics: the official organ of the International Federation of Gynaecology and Obstetrics* 1979, **17:**135-138.

12. Hardy E, Herud K: **Effectiveness of a contraceptive education program for postabortion patients in Chile.** *Studies in family planning* 1975, 6(7):188-91.

13. Hoke TH, Wheeler SB, Lynd K, Green MS, Razafindravony BH, Rasamihajamanana E, Blumenthal PD: **Community-based provision of injectable contraceptives in Madagascar: 'task shifting' to expand access to injectable contraceptives.** *Health policy and planning* 2012, **27:**52-59.

14. Kanchanasinith K, Piyapinyo P, Pitaktepsombati P, Vibulsresth S, Gates DS, Janowitz B, Robbins M: **Postpartum Sterilization by Nurse-Midwives in Thailand.** *International Family Planning Perspectives* 1990, **16:**55-58.

15. Kim KS, Arshat H, Jali AHJ, Sucm E, Suhcdmi A: **Paramedic insertion of intrauterine device in a Malaysian family planning clinic.** *Malaysian J Reprod Health* 1987, **5:**11-16.

16. Koetsawang S VS, Satayapan S, Srisupanditi S, Apimas S: **Postpartum sterilization by operating-room nurses in Thailand.** *International journal of gynaecology and obstetrics: the official organ of the International Federation of Gynaecology and Obstetrics* 1980, **19:**201-204.

17. Loghmani M, Mitra M: **Evaluation of trained midwives in a copper-T IUD insertion Program in Isfahan, Iran.** *International journal of gynaecology and obstetrics: the official organ of the International Federation of Gynaecology and Obstetrics* 1976, **14:**205-207.

18. Marangoni P, Pozo A, Faundes A: **A comparative study of T Cu-200 insertions by medical doctors and midwives.** *Estud Poblac.* 1976, 1(1):678-81.

19. Pastene L, Rivera M, Zipper J, Medel M, Thomas M: **IUD insertions by midwives: five years' experience in Santiago, Chile.** *International journal of gynaecology and obstetrics: the official organ of the International Federation of Gynaecology and Obstetrics* 1977, **15:**84-87.

20. Prata N, Gessessew A, Cartwright A, Fraser A: **Provision of injectable contraceptives in Ethiopia through community-based reproductive health agents.** *Bulletin of the World Health Organization* 2011, **89:**556-564.

21. Ramos R, Apelo RA: **A program of IUD insertions by paraprofessionals and physicians in the Philippines.** *International journal of gynaecology and obstetrics: the official organ of the International Federation of Gynaecology and Obstetrics* 1978, **16:**321-323.

22. Ronaghy HA, Zeighami B, Zeighami E, Nayeri F: **Insertion of IUDs by rural midwives in Iran.** *Public Health Rep* 1975, **90:**498-501.

23. Satyapan S, Varakamin S, Suwannus P, Chalapati S, Onthuam Y, Dusitsin N: **Postpartum tubal ligation by nurse--midwives in Thailand: a field trial.** *Studies in family planning* 1983, **14:**115-118.

24. Siswosudarmo R: **Insertion of Norplant by miswives and physicians: A comparative cohort study.** *Journal of the Medical Sciences* 1991, **23**.

25. Vaillant HW, Cummins GT, Richart RM, Barron BA: **Insertion of Lippes loop by nurse-midwives and doctors.** *British medical journal* 1968, **3:**671-673.

26. Villanueva Y, Mendoza I, Aguilar C, Rodríguez S, Vernon R: **Expansion of the Role of Nurse Auxiliaries in the Delivery of Reproductive Health Services in Honduras**. *FRONTIERS Final Report.* Population Council; Washington DC 2001.

27. Wright NH, Sujpluem C, Rosenfield AG, Varakamin S: **Nurse-midwife insertion of the copper T in Thailand: performance, acceptance, and programmatic effects.** *Studies in family planning* 1977, 8(9):237-43.

28. Zhou SW, Chi IC: **Immediate postpartum IUD insertions in a Chinese hospital--a two year follow-up.** *International journal of gynaecology and obstetrics: the official organ of the International Federation of Gynaecology and Obstetrics* 1991, **35:**157-164.
